# Beyond the pooled mean: distributional structure in the attentional-focus meta-analysis

**DOI:** 10.3389/fpsyg.2026.1916455

**Published:** 2026-09-28

**Authors:** Boyi Zeng, Xupeng Zhang, Pan Wang

**Affiliations:** 1 School of Physical Education, Xihua University, Chengdu, Sichuan, China; 2 Sichuan Jiaotong Polytechnic University, Chengdu, China

**Keywords:** attentional focus, distributionally robust optimization, effect size distributions, external focus, meta-analysis, publication bias, Wasserstein distance

## Abstract

**Introduction:**

Meta-analyses of attentional focus in motor learning and performance have focused almost exclusively on pooled mean effect sizes, leaving potential distributional features under-examined. This study evaluates whether distributional tools applied to study-level effect-size posterior mixtures can capture evidence structures—such as directionally contrary evidence and shape consistency—that conventional mean-focused meta-analytic models obscure.

**Methods:**

We reanalyzed 200 effect sizes from the published attentional-focus literature using three distributional frameworks applied to study-level effect-size posterior mixtures rather than participant-level raw distributions: Wasserstein-1 (*W*_1_) distances, a distributional heterogeneity measure *H*_*w*_, and distributionally robust optimization (DRO). Against a zero-effect benchmark, *W*_1_ was decomposed into the pooled mean and a non-negative residual equal to twice the expected negative part of the mixture, quantifying the proportion of evidence opposing the recommended instructional focus. *H*_*w*_ was compared against the location-only prediction 1−*I*^2^/100, and DRO stability margins (expressed as signal-to-noise ratios) were benchmarked against conventional trim-and-fill publication-bias corrections.

**Results:**

The directional residual derived from accounted for 36.4% of the transport distance for transfer outcomes, 20.5% for retention, and 13.5% for immediate performance; its bootstrap confidence intervals excluded zero across all evaluated outcomes, demonstrating that no sub-literature in this sample is fully summarized by its mean alone. The distributional heterogeneity measure exceeded location-only predictions across all five outcomes, most prominently for the distance effect (0.66 vs. 0.22), indicating greater agreement in distributional form than traditional variance-based indices suggest. Conversely, the outcome with the highest variance heterogeneity (*I*^2^ = 87.8% for EMG efficiency) exhibited the smallest directional residual (8.5%), as its contrary evidence lay close to the null point. DRO stability margins consistently exceeded trim-and-fill corrections across all primary outcomes.

**Discussion:**

Distributional metrics complement conventional pooled-mean comparisons by decoupling directional complexity from variance-based heterogeneity and providing robust stability bounds against data perturbation. However, because these analyses operate on study-level posterior mixtures, claims regarding individual participant-level tail behavior or extreme outcomes remain constrained and require access to raw participant-level data.

## 1 Introduction

When a physical therapist instructs a stroke patient to “focus on the floor beneath your feet” rather than “focus on your foot,” the recommendation often rests on a single number: Hedges' *g* = 0.264 for immediate performance and *g* = 0.583 for retention, as reported by Chua et al. (2021) across 73 performance and 40 retention effect sizes. Wulf (2013) and Wulf and Lewthwaite (2016) have translated these averages into coaching guidelines and textbook prescriptions: External attentional focus is superior to internal focus. However, an average tells a practitioner little about how the evidence is distributed across studies—whether effects are tightly clustered, highly dispersed, or driven by tails of unusually large or unusually small estimates. The question is whether the mean difference exists and whether the mean alone is an adequate summary of the evidence.

The external-focus advantage is one of the most replicated findings in motor learning. Wulf et al. (1998) first demonstrated that directing attention to movement effects rather than body parts improved task accuracy, and the effect has since been documented across diverse domains: Chiviacowsky et al. (2010) showed advantages for balance, Abdollahipour et al. (2015) for gymnastics, Lohse et al. (2010) for dart throwing, and Schücker et al. (2009) for running economy, in populations ranging from children (Chiviacowsky et al., 2013) to skilled athletes Porter et al., (2013). Wulf et al. (2001) and Wulf and Prinz (2001) proposed the constrained-action hypothesis to explain why: external focus promotes automatic motor control, whereas internal focus constrains the system through conscious intervention. Wulf and Lewthwaite (2016) integrated attentional focus with motivation and autonomy in the OPTIMAL theory, positioning external focus as one of three pillars that optimize performance. The empirical evidence is extensive—200 independent effect sizes across five outcome categories in the most recent systematic review—but whether its distributional structure has been fully characterized is another matter.

Meta-analysis has been the primary vehicle for summarizing this evidence. Chua et al. (2021) synthesized 73 performance studies, 40 retention studies, and 22 transfer studies, reporting statistically significant effects across all outcomes. The standard workflow—compute Hedges' *g* for each study, pool under a random-effects model, test *H*_0_: ḡ = 0, report ḡ with its confidence interval—follows conventions established by Glass (1976) and Hedges and Olkin (1985) and codified in the Higgins et al. (2019) Cochrane Handbook. Software such as Viechtbauer (2010) metafor package has made this routine. The result is a well-accepted body of evidence: external focus produces a small but reliable performance benefit and a moderate-to-large retention benefit.

However, this workflow reduces each study's findings to a single number and then averages across studies. Two distributions with identical means can have radically different tail behavior, variance, and skewness. Cohen (1994) warned against mistaking statistical significance for practical importance; Cumming (2014) advocated estimation and interval-based inference over null-hypothesis testing; (Ioannidis 2005) argued that most published findings are false; the Open Science Collaboration (2015) replication project found that only 36% of replication attempts produced statistically significant results; and Simmons et al. (2011) demonstrated that undisclosed flexibility in data analysis can produce significant *p*-values from noise. These reforms, valuable as they are, share a common premise: They remain focused on the mean. The shift from “is the effect significant?” to “how large is the effect?” does not ask what the *distribution* of the effect looks like, whether it is symmetric, whether the variance differs between conditions, or whether the tails carry information that the center does not.

A recent exchange in the attentional-focus literature crystallizes this limitation. Chua et al. (2021) reported that external focus produces significant mean effects (*g* = 0.264 for performance, *g* = 0.583 for retention). McKay et al. (2024) countered with a Bayesian selection model (3PSM) that shrinks the performance effect to *g* ≈ 0.01 after publication-bias correction. The debate is about the *center* of the distribution. Neither side asks whether the *shape* of the study-level effect-size distribution—its variance, skewness, and tail weight—carries information that the mean comparison discards. Are positive effects tightly clustered or widely dispersed? Are the tails dominated by a few extreme studies? Does the shape of the effect-size distribution vary across outcomes more than the mean alone would suggest?

These questions matter for practice. A therapist who sees an average benefit of *g* = 0.264 may still want to know whether that estimate is supported by a tight cluster of studies or by a more irregular distribution. A coach who encounters heterogeneous transfer effects may need to know whether the heterogeneity lies in the *size* of the effect (some studies find large effects, others small) or in its *shape* (some studies are tightly concentrated, others highly variable). In both cases, the mean provides an incomplete answer. Practitioners and reviewers therefore need methods that compare *entire distributions* of study effects, not just their averages.

We bring three distributional tools, developed elsewhere in statistics and operations research, to bear on this question. The first is the Wasserstein-1 distance (*W*_1_), which compares entire probability distributions rather than their means (Kantorovich, 1960; Villani, 2009; Panaretos and Zemel, 2019). For two normal distributions with the same variance, *W*_1_ reduces to the absolute mean difference; against a point-mass null, it is the expected absolute effect size, which can never fall below the absolute mean of the pooled mixture, and the excess captures distributional structure that the mean comparison discards. The second is distributional heterogeneity (*H_W_*), which extends the conventional *I*^2^ beyond location. While *I*^2^ asks whether studies disagree about the *size* of the effect, *H_W_* asks whether they disagree about the *shape* of the effect-size distribution. When *H_W_* is low, the “average effect” may not represent any particular study context well, and no single summary can adequately describe the evidence across studies. The third is distributionally robust optimization (DRO; Kuhn et al. 2019), which tests whether the external-focus recommendation survives worst-case distributional perturbations. Instead of asking “does external focus win on average?” DRO asks “how much distributional uncertainty can the recommendation withstand before it ceases to be optimal?” This reframes the publication-bias debate from “what is the corrected mean?” to “how much distributional distortion can the result absorb?”

These tools are not exotic. Wasserstein distances are a standard metric in machine learning (Peyré and Cuturi, 2019) and econometrics (Galichon, 2016), with statistical inference developed by Sommerfeld and Munk (2018) and del Barrio et al. (1999). DRO has become a cornerstone of operations research (Kuhn et al., 2019). Distributional meta-analysis is an emerging strand in statistical methodology (Mathur and VanderWeele, 2020), and a parallel movement is already pushing heterogeneity assessment beyond *I*^2^ through variance-decomposition and multi-source heterogeneity frameworks (Kepes et al., 2024; Holzmeister et al., 2024; Krefeld-Schwalb et al., 2025). Our contribution is therefore an application rather than new statistical theory: The transport metrics, the heterogeneity index, and the DRO stability analysis are imported directly from this methodological literature (Panaretos and Zemel, 2019; Kuhn et al., 2019; Mathur and VanderWeele, 2020) and adapted to study-level effect-size mixtures in the attentional-focus domain, where the synthesis debate has so far been conducted almost exclusively about means. We work from the effect-size corpus assembled by McKay et al. (2024), the most recent systematic review of this literature and the one that reports an exact standard error for every effect size, so that our conclusions are directly comparable to the most current synthesis of the debate.

Our analysis yields three contributions. First, we give the transport distance against a point-mass null an exact decomposition into the pooled mean and a non-negative residual and show that the residual has a direct substantive reading: It equals twice the expected negative part of the pooled mixture, so it measures how much of the study-level evidence points against the recommended instruction, and how far. Applied to the corpus, this residual accounts for 36.4% of the transport distance for transfer, 20.5% for retention, and 13.5% for immediate performance, with bootstrap intervals excluding zero in every case. None of these literature studies is fully described by its average.

Second, we show that this residual is not a restatement of conventional heterogeneity. Across the five outcomes, *H_W_* exceeds the location-only prediction 1−*I*^2^/100 without exception, so studies agree on distributional form more than variance-based indices imply. More pointedly, the two rankings come apart: EMG efficiency has the highest *I*^2^ in the corpus (87.8%) and the *smallest* residual (8.5%), while transfer has a lower *I*^2^ (83.5%) and by far the largest residual. The reason is instructive. EMG places 20.2% of its pooled mass below zero, more than retention or performance; but because the residual weights that mass by its depth, EMG's shallow contrary evidence contributes little while transfer's—roughly three times deeper—contributes a great deal. *I*^2^ sees only that study means are dispersed, and counting how often the evidence turns negative does not separate the two cases either: the share of pooled mass below zero orders EMG and transfer correctly but says nothing about how far apart they are, and a raw count of negative effect sizes reverses the order.

Third, we place stability margins and publication-bias corrections on a common scale. Expressed as signal-to-noise ratios, the DRO margins are ε^*^ = 0.626 (performance), 0.742 (retention), and 0.587 (transfer), each exceeding its corresponding trim-and-fill correction in the same units. Separately, comparing mean-shift and shape-distortion diagnostics shows they need not agree: Transfer has the largest trim-and-fill shift in the corpus but only a middling shape divergence, while EMG efficiency shows the reverse. Which outcome looks most bias-affected depends on which question is asked, and we argue that both should be reported.

For applied decisions, these results matter beyond the headline mean. When a therapist, coach, or trainer chooses between external and internal focus, what matters alongside the average benefit is how much of the supporting evidence points the other way, and how far. On that criterion, the transfer literature is the most equivocal of the five—over a third of its transport distance lies beyond its mean, and its contrary evidence is the deepest in the corpus—while immediate performance, despite the smaller pooled effect that has drawn most of the critical attention, is comparatively concentrated. Every pooled estimate here is positive and every prediction interval includes zero, so a recommendation defensible on average may still fail for a particular learner or task. At the same time, stronger claims about participant-level tails or catastrophic outcomes would require rawer data than the current meta-analytic summaries provide.

Section 2 reviews the attentional-focus literature and identifies the mean-centric analytical tradition that underlies its current limitations. Section 3 defines *W*_1_, *H_W_*, and DRO and describes how we apply them to the McKay et al. (2024) corpus. Section 4 presents the distributional comparisons, quantifies the scale and tail components that Hedges' *g* discards, and assesses the stability of the external-focus recommendation under worst-case perturbations. Section 5 examines the implications for theory, for the Chua–McKay debate, and for practitioners in rehabilitation and sport. Section 6 concludes.

## 2 Background

### 2.1 The attentional focus literature

The distinction between external and internal attentional focus originated in a series of experiments by Wulf et al. (1998), who demonstrated that directing learners' attention to the effects of their movements—rather than to the movements themselves—yielded more accurate and efficient performance. The theoretical account, the constrained-action hypothesis (Wulf et al., 2001; Wulf and Prinz, 2001), proposes that an external focus permits automatic motor control processes to operate without interference, whereas an internal focus engages conscious monitoring that disrupts the fluid coordination these processes provide. Critically for the present analysis, this hypothesis motivates a distributional question that the literature has not tested directly: If external focus promotes automaticity and internal focus introduces conscious interference, then the resulting distribution of observed study effects may differ in mean, dispersion, and tail behavior. The mean difference captures location; it cannot characterize the full shape of the empirical effect-size distribution.

The empirical evidence consistently supports the directional prediction. Chiviacowsky et al. (2010) showed that external focus improves balance learning in older adults, while Lohse et al. (2010) demonstrated higher accuracy in dart throwing with external cues. Abdollahipour et al. (2015) extended the finding to gymnastics and Schücker et al. (2009) to running economy. Chiviacowsky et al. (2013) found the advantage in children with intellectual disabilities, and Porter et al. (2013) confirmed it in skilled athletes. Across tasks and populations, the external-focus advantage appears in both immediate performance and longer-term retention and transfer. What these studies report, without exception, is a mean difference—an effect size that averages across participants and trials. Whether the advantage also manifests in reduced outcome variability or thinner left tails is a question the existing evidence does not address because the existing analyses were not designed to ask it.

Wulf and Lewthwaite (2016) integrated attentional focus within the broader OPTIMAL theory of motor learning, which identifies external focus as one of several conditions that promote automaticity, alongside greater expectancies and autonomy. The theory predicts that motivation and attention interact: When learners expect success and have choice, the benefits of external focus amplify. This interaction, too, has distributional implications. If motivation enhances automaticity, then one may expect higher means and more concentrated study-level effects in some contexts. The OPTIMAL theory thus deepens the theoretical expectation that the external-focus advantage may be distributional, not merely locational, even though the empirical tests have so far treated it as locational alone.

The magnitude of the empirical evidence has been quantified in several meta-analyses. Chua et al. (2021) aggregated 73 independent effect sizes for performance, 40 for retention, and 22 for transfer; their headline findings—Hedges' *g* = 0.264 for performance and *g* = 0.583 for retention—became the standard reference for coaching guidelines and textbook prescriptions. McKay et al. (2024) subsequently extended the search to 200 effect sizes across five outcome categories and reached a markedly more cautious conclusion after correcting for publication bias. These numbers answer the question “does external focus help on average?” They do not answer whether the distributional shape of the advantage—its compactness, symmetry, and tail behavior—is consistent across studies or varies in ways that a single pooled effect size obscures.

### 2.2 The mean-centric paradigm and its limitations

Meta-analysis in psychology has, since its inception, organized evidence around the mean effect size. Glass (1976) coined the term and established the enterprise of synthesizing study-level means; Hedges and Olkin (1985) and Hunter and Schmidt (1990) formalized the random-effects framework that allows the true effect to vary across studies; Cohen (1988) supplied the magnitude conventions—small, medium, large—that gave practitioners a shared language for interpreting pooled means; Borenstein et al. (2009) consolidated these advances into an authoritative textbook that has become the field's standard reference; and the Higgins et al. (2019) Cochrane Handbook codified workflow standards for systematic reviews. Software implemented the workflow: The Viechtbauer (2010) metafor package in R made it possible to compute a pooled effect size, test its significance, and produce a forest plot in a few lines of code. The dominant procedure—compute Hedges' *g* for each study, pool under a random-effects model, test *H*_0_:ḡ = 0, report ḡ with its confidence interval—is taught in every meta-analysis course and practiced in every systematic review. Its success is understandable: a single number answers the practitioner's most pressing question, and a confidence interval quantifies its precision.

This procedure has faced several waves of criticism; however, each wave has reinscribed the focus on the mean. Cohen (1994) argued that dichotomous significance testing obscures effect-size magnitude, but his remedy—report the mean more carefully—keeps the mean at the center. Cumming (2014) advocated estimation and interval-based inference over null-hypothesis testing, a shift from “is it significant?” to “how large is it?” that still treats the effect as a single number. Benjamin et al. (2018) proposed lowering the significance threshold to .005, which tightens the gate but does not ask what is being let through. The replication crisis (Ioannidis, 2005; Open Science Collaboration, 2015) exposed the fragility of mean-effect claims, and Simmons et al. (2011) showed that undisclosed analytic flexibility can manufacture significance from noise. These are serious and necessary reforms. They are also reforms that leave the distributional question unasked: Even after every reform is enacted, practitioners still know only the center of the effect-size distribution, not its shape.

Publication bias diagnostics compound the mean-centric limitation. Funnel-plot asymmetry (Egger et al., 1997) and trim-and-fill correction (Duval and Tweedie, 2000) are the standard tools for detecting and correcting selective publication, but they test whether the mean of the published distribution differs from zero—not whether the shape of the published distribution differs from the shape of the underlying one. The critiques by Sterne et al. (2000) and Lau et al. (2006) demonstrated that visual asymmetry in funnel plots can reflect genuine heterogeneity rather than bias, complicating interpretation. (Rothstein et al. 2005) provided a systematic review of these and other publication-bias diagnostics, cataloging their assumptions and failure modes. None of these diagnostics examines whether publication bias compresses variance, thickens tails, or introduces skewness that the mean alone cannot detect. The question “is the mean inflated?” is not the same as “does the shape survive?”—and the field has asked only the first.

The attentional-focus literature crystallizes this limitation. Chua et al. (2021) reported statistically significant mean effects across performance, retention, and transfer. McKay et al. (2024) countered with a Bayesian three-parameter selection model that shrinks the performance effect to *g* ≈ 0.01 after correcting for publication bias. The debate is entirely about the center of the distribution. Neither side asks whether the shape of the distribution—its variance, skewness, and tail weight—carries information that the mean comparison discards. A debate conducted entirely about the mean, using tools designed for the mean, cannot answer a distributional question. The next section introduces a set of methods that compare entire distributions rather than their averages.

### 2.3 Distributional analysis: Wasserstein distances and beyond

Two premises follow from the preceding discussion: The attentional-focus literature has theoretical reasons to expect distributional differences between external and internal focus, and the existing meta-analytic toolkit cannot detect them. Optimal transport theory provides the mathematical foundation for a remedy. The Wasserstein-1 distance (*W*_1_) between two probability distributions *P* and *Q* measures the minimum cost of transporting the probability mass of *P* into *Q* (Kantorovich, 1960; Villani, 2009; Santambrogio, 2015). If *P* and *Q* are two piles of sand, *W*_1_(*P, Q*) is the total amount of sand multiplied by the distance it must be moved to reshape *P* into *Q*. This geometric interpretation makes *W*_1_ sensitive to the entire shape of each distribution, not just its center: Two distributions that share a mean but differ in spread, skewness, or tail weight will yield *W*_1_ > 0, whereas the mean-based comparison would register no difference at all.

In the one-dimensional case—which applies directly to effect-size distributions—*W*_1_ has a closed-form expression as the integral of the absolute difference between quantile functions (Panaretos and Zemel, 2019). For simple Gaussian pairs, *W*_1_ can be read in terms of location and scale differences. In our application, however, the relevant objects are mixture posteriors of study-level effects, so the transport summaries are descriptive rather than a direct closed-form restatement of Hedges' *g*. This is what makes *W*_1_ informative for meta-analysis: It allows us to ask whether the pooled evidence differs only in average magnitude or also in overall shape.

Several alternative distributional metrics exist, but each carries limitations that *W*_1_ avoids. Kullback and Leibler (1951) defined the Kullback-Leibler divergence to measure information loss when one distribution is used to approximate another, but it requires absolutely continuous distributions with shared support—it is undefined when *P* and *Q* have non-overlapping regions, a situation that arises when one distribution has a thinner tail than the other. The Cramér distance, related to the dispersion orderings studied by Bickel and Lehmann (1976), measures the *L*^2^ distance between distribution functions but lacks the natural decomposition into location and shape that *W*_1_ provides. The Jensen-Shannon divergence, a symmetrized and smoothed variant of the KL divergence, resolves the asymmetry problem but still imposes smoothness assumptions that need not hold for empirical effect-size distributions. Peyré and Cuturi (2019) surveyed these alternatives and noted that the Wasserstein distance uniquely combines metric properties, computational tractability, and interpretability: A practitioner can understand “the external-focus distribution is shifted right by this much and compressed by this much” without specialized mathematical training because the quantile-based decomposition maps directly onto the substantive questions that practitioners ask.

Statistical inference for *W*_1_ has been developed by del Barrio et al. (1999), who established asymptotic distributions and confidence intervals for one-sample goodness-of-fit tests, and by Sommerfeld and Munk (2018), who extended the framework to two-sample comparisons on finite spaces. The Wasserstein barycenter (Cuturi and Doucet, 2014)—the consensus distribution that minimizes average *W*_1_ to all study-level distributions—provides a natural analog to the pooled effect size, but for entire distributions rather than point estimates. Computational frameworks are well established: Peyré and Cuturi (2019) provided a detailed treatment of optimal transport algorithms, and Galichon (2016) surveyed applications in economics and matching markets. In psychology meta-analysis, distributional methods remain rare. Mathur and VanderWeele (2020) introduced distributional metrics for replication assessment, establishing that comparing entire distributions rather than point estimates can reveal when a replication fails in shape rather than in location. A parallel development outside optimal transport is now pushing heterogeneity assessment beyond *I*^2^ from a different direction. Kepes et al. (2024) audited heterogeneity reporting across five leading journals, found that most syntheses report but rarely interpret heterogeneity statistics thoroughly, and recommended routine reporting of prediction intervals and absolute heterogeneity measures. Holzmeister et al. (2024) provided a framework that partitions heterogeneity in effect-size estimates into population, design, and analytical sources, estimated from multilab replications, prospective meta-analyses, and multianalyst studies; Krefeld-Schwalb et al. (2025) added the caution that estimates of population heterogeneity are themselves constrained by how heterogeneous the sampled populations are. These frameworks decompose the *sources* of variation in effect sizes; the transport-based approach taken here is complementary, decomposing the *geometry* of the pooled evidence—location versus shape—for a fixed set of studies. We extend this direction to the attentional-focus domain, adding distributional heterogeneity (*H_W_*) and distributionally robust optimization (DRO) as complementary tools that address the specific questions the mean-based debate cannot: whether the external-focus advantage is distributional rather than merely locational, whether heterogeneity lies in effect shape as well as effect size, and whether the recommendation survives distributional uncertainty that a single corrected mean cannot capture.

## 3 Method

### 3.1 Data sources

We reanalyze the effect-size corpus compiled by McKay et al. (2024), whose systematic review extends the earlier synthesis of Chua et al. (2021) and is distributed as a machine-readable dataset with an exact standard error for every effect size. Both reviews followed PRISMA guidelines and included studies that compared external-focus and internal-focus instructions in motor tasks, using Hedges' *g* (Hedges, 1981) as the effect-size metric; positive values indicate an external-focus advantage. The corpus contains 200 independent effect sizes across five outcome categories: immediate performance (*k* = 100, *N* = 2, 182), retention (*k* = 49, *N* = 1, 614), transfer (*k* = 23, *N* = 797), EMG efficiency (*k* = 16, *N* = 299), and the distance effect (*k* = 12, *N* = 287). Studies span diverse tasks (balance, golf, darts, sprinting, and gymnastics) and populations (children, healthy adults, older adults, clinical patients, and elite athletes). The complete study-level records are reproduced in the Supplementary material.

We use this corpus rather than the smaller Chua et al. (2021) tables for two reasons. First, it is the more recent and more inclusive systematic review of the same literature. Second, and decisively for a distributional analysis, it reports an exact standard error for each effect size, so the study-level posteriors that our transport summaries depend on can be constructed without approximation. Because our pooled estimates are therefore computed from a larger corpus than the one Chua et al. (2021) analyzed, they differ from their published summaries; where we compare our results to theirs, we cite their reported values explicitly rather than recomputing them.

McKay et al. (2024) also applied a three-parameter selection model (3PSM; Vevea and Hedges 1995) to correct for publication bias. The 3PSM estimates the probability that a study with a non-significant result remains unpublished and adjusts the pooled effect accordingly. Their corrected estimates, together with the trim-and-fill correction we compute directly from the same data (Duval and Tweedie, 2000), provide the comparison points for our distributionally robust optimization (DRO) analysis: If the external-focus recommendation survives the worst-case perturbation implied by a correction, the recommendation is stable under that model of publication bias.

Study-level standard errors are taken directly from the source dataset, which reports an exact value for all 200 effect sizes; no approximation is used anywhere in the analysis. This matters because a widely used shortcut, $s_{i}\approx \sqrt{4/n_{i}}$, discards the effect-size-dependent term in the standard error of Hedges' *g* (Hedges, 1981),


$$\begin{array}{l} s_{i}=\sqrt{\frac{n_{1i}+n_{2i}}{n_{1i}n_{2i}}+\frac{g_{i}^{2}}{2\left(n_{1i}+n_{2i}\right)}}, \end{array}$$ (1)

and therefore systematically understates the uncertainty of studies with large effects. Because that shortcut appears in applied reanalyses of this literature, Section 4.7 reports what it would have done to our transport summaries had we used it. Under Equation 1 the standard error grows with |*g*_*i*_|, so the truncated form narrows the posteriors of exactly those studies that sit farthest from the null, and it distorts the pooled mixture in a direction that inflates apparent distributional concentration.

### 3.2 Analytic overview

Our analysis proceeds in four stages, each addressing a question that the conventional mean-based workflow cannot. First, we summarize the pooled study-level effect-size posteriors with Wasserstein-1 distance (*W*_1_), separating a transport-location component from a non-negative residual that measures how much of the study-level evidence lies below the null, and how far. Second, we compute distributional heterogeneity (*H_W_*), which extends the conventional *I*^2^ of Higgins and Thompson (2002) beyond location disagreement to detect whether studies also disagree about the shape of the effect-size distribution. Third, we apply distributionally robust optimization (DRO) to test whether the external-focus recommendation remains optimal under worst-case perturbational shifts of magnitude ε; the largest ε at which the recommendation still holds (ε^*^) is the stability margin. Fourth, we measure the Kullback-Leibler (KL) divergence between the published and trim-and-fill-corrected effect-size mixtures to determine whether publication bias materially alters distributional shape. These four analyses are complementary: *W*_1_ summarizes distance, *H_W_* summarizes agreement, DRO summarizes stability, and KL summarizes distortion. Figure 1 summarizes the workflow.

**Figure 1:**
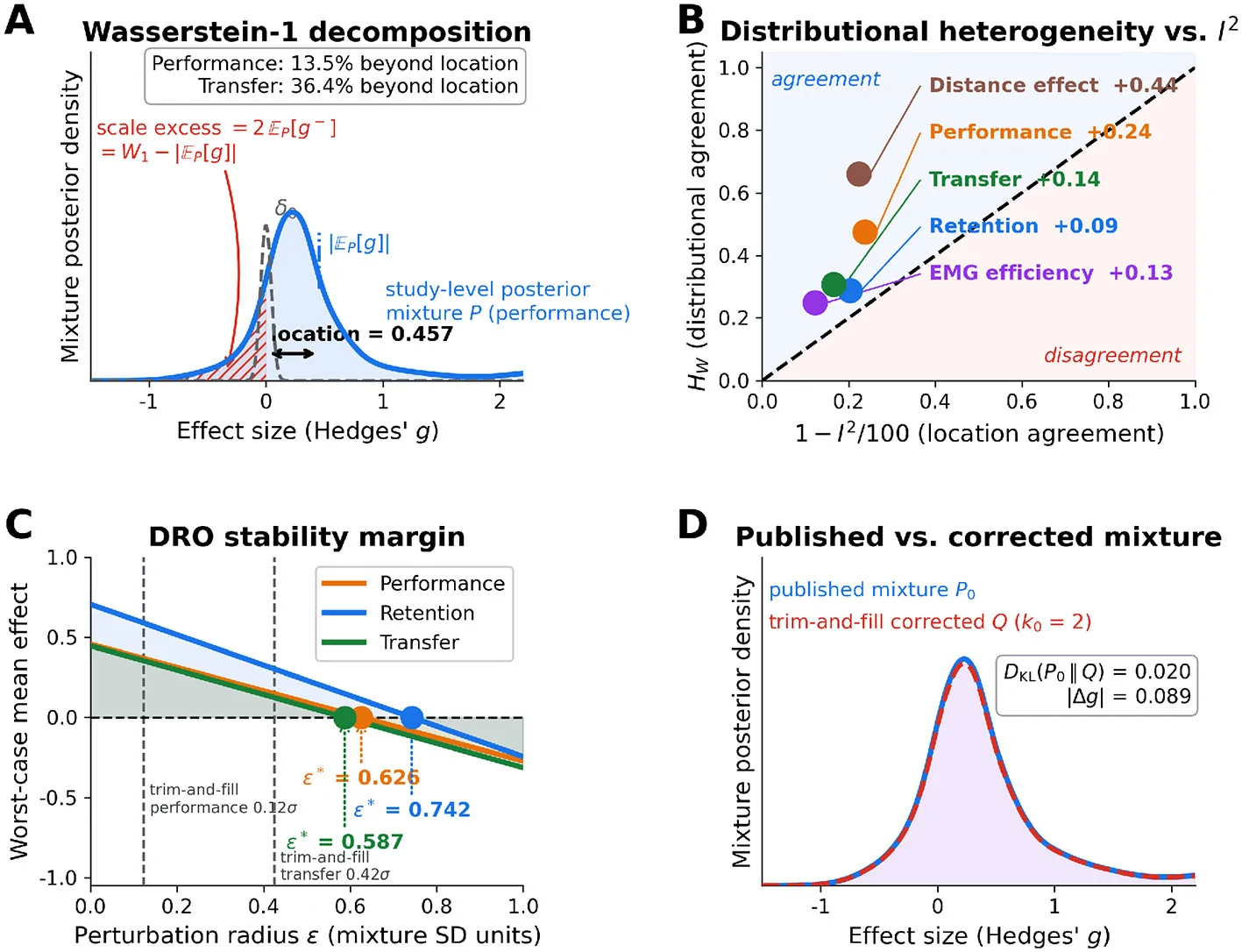
Overview of the distributional analysis pipeline. Every plotted curve and annotated value is read from the analysis output file that also generates Tables 1, 2. **(A)** decomposes the transport distance for the performance mixture: The pooled study-level posterior mixture is transported to the zero-effect benchmark δ_0_, and *W*_1_ separates into a location term |𝔼_*P*_[*g*]| = 0.457 and a non-negative scale excess equal to $2{𝔼}_{P}\left[g^{-}\right]$ (hatched), which is 13.5% of *W*_1_ for performance and 36.4% for transfer. **(B)** plots distributional heterogeneity *H_W_* against the location-only prediction 1−*I*^2^/100 for all five outcomes; each point lies above the diagonal, and the annotated figure is the divergence *H_W_* − (1 − *I*^2^/100). **(C)** shows the DRO worst-case mean effect 𝔼_*P*_[*g*]−εσ_*P*_ as a function of the perturbation radius ε; each curve crosses zero at its stability margin ε^*^ (0.626 for performance, 0.742 for retention, and 0.587 for transfer), and the dashed vertical lines mark the trim-and-fill corrections for performance (0.12σ) and transfer (0.42σ), expressed in the same mixture standard-deviation units. **(D)** compares the published performance mixture *P*_0_ with its trim-and-fill-corrected counterpart *Q* (*k*_0_ = 2): The two remain close in shape (*D*_KL_ = 0.020) even though the imputation shifts the pooled estimate by |Δ*g*| = 0.089.

### 3.3 Wasserstein-1 distance: comparing entire distributions

#### 3.3.1 Intuitive motivation

Consider a set of meta-analytic effect sizes for one outcome. Hedges' *g* tells us the pooled average effect, but it does not describe whether the study estimates are tightly clustered, broadly dispersed, or asymmetric. The Wasserstein-1 distance (*W*_1_) helps summarize those broader features by measuring how much probability mass must be moved, and how far, to transform one distribution into another. It is sometimes called the “earth mover's distance”: imagine *P* as a pile of sand and *Q* as the target shape; *W*_1_ is the total cost (mass × distance) of the reshaping.

For simple normal pairs, *W*_1_ can be related to differences in means and spreads. In the present study, however, the empirical objects are mixture posteriors built from study-level effect-size estimates, and the comparison distribution is a point mass at zero. In that setting, the transport distance admits an exact and interpretable decomposition, which we now state.

#### 3.3.2 Formal definition and estimation

We model the posterior distribution of the true effect for study *i* as $P_{i}=N\left(g_{i},s_{i}^{2}\right)$, where *g*_*i*_ is the observed Hedges' *g* and *s*_*i*_ is its standard error. This normal posterior is a natural choice given that, as Hedges (1981) showed, Hedges' *g* is approximately normal for moderate sample sizes. For each outcome category, we then form a pooled mixture of study-level posteriors, weighting by sample size, As shown in Equation 2:


$$\begin{array}{l} P_{\text{outcome}}=\underset{i}{\sum }w_{i}P_{i} \end{array}$$ (2)

where $w_{i}=n_{i}/\underset{j}{\sum }n_{j}$. Sample-size weighting gives larger studies proportionally greater influence on the pooled distribution, consistent with the standard practice in random-effects meta-analysis (DerSimonian and Laird, 1986; Viechtbauer, 2010). We then compute a Wasserstein summary of this pooled posterior relative to a zero-effect benchmark via the quantile representation:


$$\begin{array}{l} W_{1}\left(P_{\text{outcome}},\delta_{0}\right)=\int_{0}^{1}|F_{\text{outcome}}^{-1}\left(u\right)-F_{\delta_{0}}^{-1}\left(u\right)|du \end{array}$$ (3)

where *F*^−1^ denotes the quantile function, and δ_0_ is a point mass at zero. Because the quantile function of δ_0_ is identically zero, Equation 3 simplifies to *W*_1_(*P*_outcome_, δ_0_) = 𝔼_*P*_|*g*|, the expected absolute effect size under the pooled mixture.

#### 3.3.3 Location–scale decomposition

We split the transport distance into the part that a pooled mean already conveys and the part that it does not:


$$\begin{array}{l} \underset{W_{1}\left(P,\delta_{0}\right)}{\underset{︸}{{𝔼}_{P}|g|}}=\underset{\text{location}}{\underset{︸}{\left|{𝔼}_{P}\left[g\right]\right|}}+\underset{\text{scale excess}}{\underset{︸}{\left({𝔼}_{P}|g|-\left|{𝔼}_{P}\left[g\right]\right|\right)}}. \end{array}$$ (4)

The location term is the absolute mean of the pooled mixture—the quantity a conventional meta-analysis reports. The scale excess is the residual. Two properties make this decomposition well behaved and give the analysis its interpretive content.

**Proposition 1**. *For any pooled mixture *P* with finite first moment, the scale excess is non-negative*,


$$\begin{array}{l} {𝔼}_{P}|g|-\left|{𝔼}_{P}\left[g\right]\right|=2{𝔼}_{P}\left[g^{-}\right]\geq 0,\;g^{-}:=\max\left(-g,0\right), \end{array}$$ (5)

*with equality if and only if *P* places no probability mass below zero. Consequently *W*_1_(*P*, δ_0_) ≥ |𝔼_*P*_[*g*]| always, so the decomposition of Equation 4 never assigns a negative value to either component*.

*Proof*: Write *g* = *g*^+^ − *g*^−^ with *g*^+^ = max(*g*, 0) and *g*^−^ = max(−*g*, 0), so that |*g*| = *g*^+^ + *g*^−^. Taking expectations, ${𝔼}_{P}|g|={𝔼}_{P}\left[g^{+}\right]+{𝔼}_{P}\left[g^{-}\right]$ and ${𝔼}_{P}\left[g\right]={𝔼}_{P}\left[g^{+}\right]-{𝔼}_{P}\left[g^{-}\right]$. When the pooled mean is non-negative, $\left|{𝔼}_{P}\left[g\right]\right|={𝔼}_{P}\left[g^{+}\right]-{𝔼}_{P}\left[g^{-}\right]$, and subtracting gives ${𝔼}_{P}|g|-\left|{𝔼}_{P}\left[g\right]\right|=2{𝔼}_{P}\left[g^{-}\right]$. Since *g*^−^ ≥ 0 pointwise, the difference is non-negative, and it vanishes exactly when ${𝔼}_{P}\left[g^{-}\right]=0$, that is, when *P*(*g* < 0) = 0. The symmetric argument applies when the pooled mean is negative, with *g*^+^ in place of *g*^−^. The final inequality restates Jensen's inequality for the convex map *g*↦|*g*|.     □

The bound is stated against |𝔼_*P*_[*g*]|, the absolute mean of the pooled mixture, and not against the random-effects estimate ḡ. The two are distinct quantities—ḡ weights studies by inverse variance, the mixture by sample size—and no inequality between *W*_1_ and ḡ follows from the argument above, although in the present corpus *W*_1_ happens to exceed both.

Equation 5 gives the scale excess a concrete meaning: It is twice the probability mass that the pooled study-level evidence places on the wrong side of zero, weighted by how far onto that side it falls. A literature whose studies all point the same way, with posteriors tight enough that essentially none of the mixture crosses zero, has a scale excess of zero and is fully summarized by its mean. A literature in which some studies estimate a reversed effect, or in which small studies carry posteriors wide enough to straddle zero, has a positive scale excess, and the size of that excess measures how much the pooled mean omits. We report the excess both in absolute terms and as a share of *W*_1_.

For Gaussian study posteriors, both terms have closed forms, so no numerical integration error enters the reported values, As shown in Equation 6:


$$\begin{array}{r} {𝔼}_{P}|g|=\underset{i}{\sum }w_{i}\left[s_{i}\sqrt{\frac{2}{\pi }}e^{-g_{i}^{2}/\left(2s_{i}^{2}\right)}+g_{i}\text{erf}\left(\frac{g_{i}}{s_{i}\sqrt{2}}\right)\right],\\ {𝔼}_{P}\left[g\right]=\underset{i}{\sum }w_{i}g_{i}. \end{array}$$ (6)

We obtain 95% confidence intervals by non-parametric bootstrap over studies (*B* = 2000 replications; Efron and Tibshirani 1993), resampling the set of study-level distributions with replacement and recomputing both components at each iteration.

### 3.4 Distributional heterogeneity: *H_W_*

#### 3.4.1 Intuitive motivation

Consider two transfer studies. Study A finds a large, consistent external-focus benefit: The effect-size distribution is tall and narrow, centered well above zero. Study B finds a small, variable effect: The distribution is short and wide, with considerable probability mass below zero. These two studies disagree about the shape of the effect, not merely about its size—yet the conventional *I*^2^ of Higgins and Thompson (2002) treats both as “high heterogeneity” without distinguishing whether the disagreement lies in location (different means) or in shape (different variances, skewness, tail weight). *I*^2^ detects that studies disagree; *H_W_* detects *how* they disagree.

*H_W_* measures the agreement among study-level distributions as whole shapes. It compares two summaries: the naive pooled distribution (all posteriors concatenated) and the Wasserstein barycenter—the consensus shape that minimizes average transport cost to every study. When all studies share a similar shape, the barycenter represents them well and *H_W_* approaches 1; when studies disagree in shape, no single summary fits well and *H_W_* drops toward 0. Unlike *I*^2^, which is sensitive only to location differences, *H_W_* detects shape disagreement even when the means coincide.

#### 3.4.2 Formal definition

Let *P*_bary_ be the Wasserstein barycenter of the study-level posteriors {*P*_1_, …, *P*_*k*_}—the distribution whose quantile function is the precision-weighted average of the study quantile functions (Cuturi and Doucet, 2014). Let *P*_pooled_ be the naive pooled distribution (all posterior samples concatenated). Distributional heterogeneity is defined as


$$\begin{array}{l} H_{W}=1-\frac{\sum_{i}w_{i}\cdot W_{1}\left(P_{i},P_{\text{bary}}\right)}{\sum_{i}w_{i}\cdot W_{1}\left(P_{i},P_{\text{pooled}}\right)} \end{array}$$ (7)

where *w*_*i*_ are precision weights ($w_{i}\propto 1/s_{i}^{2}$). The numerator measures how far studies are from the consensus shape; the denominator measures how far they are from the naive pool. Precision weighting is used here (rather than the sample-size weighting adopted for *W*_1_) because *H_W_* is a measure of agreement among distributions, and more precise estimates should carry greater influence in defining the consensus shape. When the barycenter provides a much better summary than the pool—because studies agree on shape—*H_W_* approaches 1. When studies are so dispersed that even the optimal consensus shape is far from each study, *H_W_* approaches 0.

#### 3.4.3 Relation to Fréchet variance and classical heterogeneity

The measure *H_W_* is not an ad hoc construction but a special case of the Fréchet coefficient of determination in the Wasserstein metric space. Fréchet (1948) defined the mean and variance of a random object in a metric space as the point and value that minimize expected squared distance. Petersen and Müller (2019) extended this framework to regression for distributional objects, and Dubey and Müller (2019) developed a Fréchet analysis-of-variance decomposition. In this framework, the total Fréchet variance of {*P*_1_, …, *P*_*k*_} relative to a reference *R* is $V_{\text{total}}=\underset{i}{\sum }w_{i}\cdot d\left(P_{i},R\right)$, and a natural R^2^-like quantity is 1−*V*_within_/*V*_total_, where *V*_within_ uses the Fréchet mean (barycenter) as the reference. Equation 7 instantiates exactly this construction with *d* = *W*_1_ and *R* = *P*_pooled_, so *H_W_* is the Fréchet R^2^ in Wasserstein space.

This connection clarifies why *H_W_* generalizes *I*^2^. The conventional *I*^2^ of Higgins and Thompson (2002) is itself an R^2^-like ratio of between-study to total variance, but it is restricted to location disagreement. The measure *H_W_* replaces Euclidean variance with Wasserstein transport cost, capturing shape disagreement and location disagreement. The following proposition makes the correspondence precise.

**Proposition 2**. *If all study-level posteriors are Gaussian with equal variance, $P_{i}=N\left(\mu_{i},\sigma^{2}\right)$ for all *i*, then transport from each study to the barycenter is purely locational:*


$$\begin{array}{l} W_{1}\left(P_{i},P_{\text{bary}}\right)=|\mu_{i}-\bar{\mu }|, \end{array}$$ (8)

*where $\bar{\mu }=\underset{i}{\sum }w_{i}\mu_{i}/\underset{i}{\sum }w_{i}$ is the precision-weighted mean. Consequently, *H_W_* = 1 if and only if all μ_*i*_ are equal—equivalently, if and only if *I*^2^ = 0*.

*Proof*: For Gaussian posteriors $P_{i}=N\left(\mu_{i},\sigma^{2}\right)$, the cumulative distribution function (CDF) of each study is given by *F*_*i*_(*x*) = Φ((*x* − μ_*i*_)/σ), and the corresponding quantile function (inverse CDF) is shown in Equation 9:


$$\begin{array}{l} F_{i}^{-1}\left(u\right)=\mu_{i}+\sigma \Phi^{-1}\left(u\right)\text{ for }u\in \left(0,1\right), \end{array}$$ (9)

where Φ^−1^(*u*) is the quantile function of the standard normal distribution N(0,1).

By definition, the quantile function of the precision-weighted Wasserstein barycenter *P*_bary_ is the precision-weighted average of the study-level quantile functions:


$$\begin{array}{l} F_{\text{bary}}^{-1}\left(u\right)=\overset{k}{\underset{i=1}{\sum }}w_{i}F_{i}^{-1}\left(u\right), \end{array}$$ (10)

where $w_{i}=\frac{1/s_{i}^{2}}{\overset{k}{\underset{j=1}{\sum }}1/s_{j}^{2}}$ are the normalized precision weights satisfying $\overset{k}{\underset{i=1}{\sum }}w_{i}=1$. Because all study-level posteriors have equal variance ($s_{i}^{2}=\sigma^{2}$ for all *i*), the weights simplify to *w*_*i*_ = 1/*k*. Substituting the individual study quantile functions yields


$$\begin{array}{r} F_{\text{bary}}^{-1}\left(u\right)=\overset{k}{\underset{i=1}{\sum }}w_{i}\left(\mu_{i}+\sigma \Phi^{-1}\left(u\right)\right)=\left(\overset{k}{\underset{i=1}{\sum }}w_{i}\mu_{i}\right)\\ +\sigma \Phi^{-1}\left(u\right)\overset{k}{\underset{i=1}{\sum }}w_{i}=\bar{\mu }+\sigma \Phi^{-1}\left(u\right), \end{array}$$ (11)

where $\bar{\mu }=\overset{k}{\underset{i=1}{\sum }}w_{i}\mu_{i}$ is the precision-weighted mean. This shows that the Wasserstein barycenter is exactly the Gaussian distribution $P_{\text{bary}}=N\left(\bar{\mu },\sigma^{2}\right)$.

Using the quantile representation of the Wasserstein-1 distance, the transport cost between any study posterior *P*_*i*_ and the barycenter *P*_bary_ is


$$\begin{array}{l} W_{1}\left(P_{i},P_{\text{bary}}\right)=\int_{0}^{1}|F_{i}^{-1}\left(u\right)-F_{\text{bary}}^{-1}\left(u\right)|du \end{array}$$ (12)

Substituting the expressions for $F_{i}^{-1}\left(u\right)$ and $F_{\text{bary}}^{-1}\left(u\right)$:


$$\begin{array}{r} W_{1}\left(P_{i},P_{\text{bary}}\right)=\int_{0}^{1}|\left(\mu_{i}+\sigma \Phi^{-1}\left(u\right)\right)-\left(\bar{\mu }+\sigma \Phi^{-1}\left(u\right)\right)|\\ du=\int_{0}^{1}|\mu_{i}-\bar{\mu }|du=|\mu_{i}-\bar{\mu }|, \end{array}$$ (13)

which establishes Equation 8 and shows that transport is purely locational.

Next, we establish the connection to Higgins and Thompson's classical heterogeneity index *I*^2^. Recall the definition of the distributional heterogeneity measure *H_W_* from Equation 7:


$$\begin{array}{l} H_{W}=1-\frac{\sum_{i=1}^{k}w_{i}W_{1}\left(P_{i},P_{\text{bary}}\right)}{\sum_{i=1}^{k}w_{i}W_{1}\left(P_{i},P_{\text{pooled}}\right)} \end{array}$$ (14)

Since all transport distances and weights are non-negative, *H_W_* ≤ 1. Therefore, *H_W_* = 1 if and only if the numerator vanishes:


$$\begin{array}{l} \overset{k}{\underset{i=1}{\sum }}w_{i}W_{1}\left(P_{i},P_{\text{bary}}\right)=0\;\Leftrightarrow \;\overset{k}{\underset{i=1}{\sum }}w_{i}|\mu_{i}-\bar{\mu }|=0 \end{array}$$ (15)

Because the weights are strictly positive (*w*_*i*_ > 0) and the absolute differences are non-negative ($|\mu_{i}-\bar{\mu }|\geq 0$), this sum is zero if and as shown in Equation 16:


$$\begin{array}{l} |\mu_{i}-\bar{\mu }|=0\text{ for all }i=1,\ldots ,k\;\Leftrightarrow \;\mu_{1}=\mu_{2}=\cdots =\mu_{k}=\bar{\mu }. \end{array}$$ (16)

In classical random-effects meta-analysis, the true study means are modeled as drawing from a distribution with between-study variance τ^2^. The condition that all true study means are identical ($\mu_{i}=\bar{\mu }$ for all *i*) corresponds to zero between-study heterogeneity, τ^2^ = 0. Since $I^{2}=\frac{\tau^{2}}{\tau^{2}+\sigma^{2}}$, it follows directly that *I*^2^ = 0 if and only if τ^2^ = 0. Hence, *H_W_* = 1 if and only if all μ_*i*_ are equal, which is mathematically equivalent to *I*^2^ = 0.

When studies differ in variance or shape, *H_W_* diverges from 1−*I*^2^/100, and the direction and magnitude of the divergence are informative: *H_W_* > 1−*I*^2^/100 indicates that studies agree on shape more than location heterogeneity alone would predict (the barycenter captures the common form well); *H_W_* < 1−*I*^2^/100 would indicate shape disagreement beyond what location heterogeneity alone would predict.

### 3.5 Distributionally robust optimization (DRO)

#### 3.5.1 Intuitive Motivation

The standard recommendation—“use external focus because ḡ > 0”—optimizes the average outcome. But a coach choosing instructions for a competition, or a therapist prescribing cues for a frail patient, cares about more than the average. If external focus produces large gains in most studies but occasional harm in a few, a risk-averse practitioner might prefer a safer strategy. DRO formalizes this concern: instead of asking “which strategy works best on average?” it asks “which strategy remains best *even in the worst plausible version of reality*?” The “worst plausible version” is defined by a Wasserstein ambiguity ball of radius ε around the observed data—any distribution within transport distance ε of the empirical distribution is considered plausible, and the analysis optimizes against the worst case among all such distributions.

#### 3.5.2 Formal definition

For a given outcome category with pooled effect-size mixture $\hat{P}$ and point-mass null δ_0_, the DRO-optimal decision tests whether the external-focus recommendation remains preferred under the worst-case perturbation of $\hat{P}$ within a Wasserstein ball of radius ε. Formally, we compute the largest ε such that


$$\begin{array}{l} \underset{P:W_{1}\left(P,\hat{P}\right)\leq \varepsilon }{\inf}{𝔼}_{P}\left[g\right]>0 \end{array}$$ (17)

where $\hat{P}$ is the observed mixture posterior, and ε parameterizes the ambiguity set around it. For one-dimensional effect sizes, this problem admits a closed-form solution: the infimum in Equation 17 is positive if and only if


$$\begin{array}{l} {𝔼}_{\hat{P}}\left[g\right]>\varepsilon \sigma_{\hat{P}},\text{ so that }\varepsilon^{*}=\frac{{𝔼}_{\hat{P}}\left[g\right]}{\sigma_{\hat{P}}}, \end{array}$$ (18)

where $\sigma_{\hat{P}}$ is the standard deviation of the pooled mixture (Esfahani and Kuhn, 2018; Kuhn et al., 2019). We state this plainly because it bounds what the analysis can claim: In the univariate case the stability margin is the signal-to-noise ratio of the pooled evidence, and we claim no methodological novelty for the computation itself. What the DRO framing adds is a common scale. Because ε^*^ is measured in mixture standard deviations, a publication-bias correction of magnitude Δ*g* becomes comparable to it once expressed in the same units, as $\Delta g/\sigma_{\hat{P}}$. The recommendation is stable against a given correction when $\varepsilon^{*}>\Delta g/\sigma_{\hat{P}}$, equivalently when ${𝔼}_{\hat{P}}\left[g\right]$ exceeds Δ*g*—but the ratio form makes explicit how much of the margin is genuine signal and how much is an artifact of a narrow mixture. This reframes the publication-bias debate: rather than asking only “what is the corrected mean?”, DRO asks “how much distributional uncertainty can the result absorb, relative to the spread of the evidence itself?” Several caveats apply. First, the DRO analysis treats the pooled mixture posterior as the reference distribution; perturbations are to this study-level summary, not to participant-level outcome distributions. Second, the Wasserstein ball around $\hat{P}$ defines a set of distributions that are nearby in transport distance, which is a weaker form of stability guarantee than verification against every possible data-generating process. Third, because ε^*^ is defined relative to the point-mass null, it does not directly test a comparison between external-focus and internal-focus conditions; rather, it tests whether the pooled evidence for external focus survives perturbation. With these caveats, DRO provides a principled framework for assessing recommendation stability under distributional uncertainty.

### 3.6 Publication bias as distributional asymmetry

#### 3.6.1 Intuitive motivation

A funnel plot is a visual check for publication bias: If small studies with small effects are missing, the plot looks asymmetric. Egger et al. (1997) quantified this asymmetry with a regression test, and Duval and Tweedie (2000) developed the trim-and-fill procedure to correct for it—but both focus on whether the mean is inflated. They do not ask whether the *shape* of the published distribution differs from the shape of the bias-corrected one. A distribution can be right-skewed by selective publication (the left tail is missing), while the mean remains nearly unchanged; in that case, Egger's test may detect bias but cannot describe what the bias has done to the distribution. Kullback and Leibler (1951) introduced the KL divergence to measure exactly this: how much information is lost when one uses the published distribution as a proxy for the bias-corrected one.

#### 3.6.2 Formal definition

The KL divergence between the observed and bias-corrected distributions is Equation 19:


$$\begin{array}{l} D_{\text{KL}}\left(P_{\text{obs}}\|P_{\text{corrected}}\right)=\int p_{\text{obs}}\left(x\right)\log\frac{p_{\text{obs}}\left(x\right)}{p_{\text{corrected}}\left(x\right)}dx \end{array}$$ (19)

Unlike Egger's test, which produces a *p*-value for the null hypothesis of no mean inflation, KL divergence is continuous and shape-sensitive: It can detect that the left tail is missing even when the mean is unaffected. We estimate *D*_KL_ by comparing the observed posterior mixture with a bias-corrected mixture constructed via the trim-and-fill algorithm of Duval and Tweedie (2000), which imputes missing studies to restore funnel-plot symmetry. When *D*_KL_ ≈ 0, the published and corrected distributions are nearly indistinguishable in shape, and the publication-bias debate is a debate about prior assumptions rather than about observed data.

## 4 Results

### 4.1 Descriptive overview of the data

We begin with the conventional random-effects summaries, computed with the DerSimonian–Laird estimator (DerSimonian and Laird, 1986) over the full corpus. Immediate performance yields a pooled Hedges' *g* of 0.379 (95% CI [0.294, 0.465], *k* = 100, *N* = 2, 182, τ^2^ = 0.129, *Q*(99) = 417.3, *I*^2^ = 76.3%). Retention shows a larger effect, *g* = 0.680 (95% CI [0.463, 0.896], *k* = 49, *N* = 1, 614, τ^2^ = 0.450, *Q*(48) = 236.4, *I*^2^ = 79.7%), and transfer *g* = 0.506 (95% CI [0.238, 0.774], *k* = 23, *N* = 797, τ^2^ = 0.331, *Q*(22) = 133.3, *I*^2^ = 83.5%). EMG efficiency gives *g* = 0.751 (95% CI [0.414, 1.087], *k* = 16, *N* = 299, τ^2^ = 0.388, *Q*(15) = 123.0, *I*^2^ = 87.8%) and the distance effect *g* = 0.318 (95% CI [0.043, 0.593], *k* = 12, *N* = 287, τ^2^ = 0.165, *Q*(11) = 49.2, *I*^2^ = 77.6%).

Two features of this table deserve comment before the distributional analysis begins. First, every pooled estimate excludes zero, so on the conventional reading the external-focus advantage is present in all five outcomes. Second, and less reassuringly, heterogeneity is uniformly high: *I*^2^ ranges from 76.3% to 87.8%, and every 95% prediction interval includes zero (performance [−0.33, 1.09]; retention [−0.65, 2.01]; transfer [−0.65, 1.66]; EMG [−0.52, 2.02]; distance [−0.52, 1.16]). A practitioner drawing a study at random from any of these literature studies therefore has no assurance of a positive effect, even though the pooled mean is reliably positive in all five. This is the gap between an average and a distribution that the analyses below are designed to quantify. It also differs from the picture in the earlier and smaller synthesis of Chua et al. (2021), who reported low heterogeneity for immediate performance (*I*^2^ = 13.7%); on the larger corpus, the performance literature is as heterogeneous as the others.

These central tendencies and between-study variances, however, leave the *distributional* structure behind these means unexamined. Two outcomes with identical pooled means and conventional heterogeneities can nevertheless have very different distributional shapes: one concentrated around the mean, and the other spread across a wide range with asymmetric tails. Conventional meta-analytic summaries cannot distinguish these cases because they do not characterize the shape of the mixture distribution that emerges when study-level posteriors are pooled. The four analyses that follow (Wasserstein distance, distributional heterogeneity, distributionally robust optimization, and KL divergence) each reveal a different facet of this structure.

### 4.2 Study-level characteristics: an integrated view

All 200 study-level records—effect size, exact standard error, sample size, task, and risk-of-bias rating—are reproduced in the Supplementary material. Following Higgins and Thompson (2002), we regard transparent reporting of study-level data as essential for any meta-analytic claim, and the distributional summaries below are computed from these records rather than from the published pooled estimates.

Two features of the raw records shape everything that follows. First, the *spread* of precision is wide: Standard errors in the performance corpus range from 0.085 to 0.991, so the least precise studies contribute posteriors an order of magnitude broader than the most precise. This heteroscedasticity is not a nuisance to be averaged away; it determines how much of the pooled mixture spills across zero, which by Proposition 1 is exactly what the scale excess measures. Second, the *direction* of effects is not uniform: 14 of the 100 performance effect sizes are negative, as are 7 of 49 retention and 7 of 23 transfer effect sizes, and the 95% intervals of many more cross zero. As Cumming (2012) emphasized, overlapping confidence intervals do not imply the absence of a real effect—but they do indicate that the pooled effect-size distribution places non-trivial probability mass below zero, a feature that any complete analysis must acknowledge and that a pooled mean by construction cannot report.

Figure 2a Panel B makes the point concretely for the performance corpus: The individual estimates fan out from −0.55 to 4.22, and although the pooled mean sits at 0.379, roughly one study in seven points the other way.

**Figure 2:**
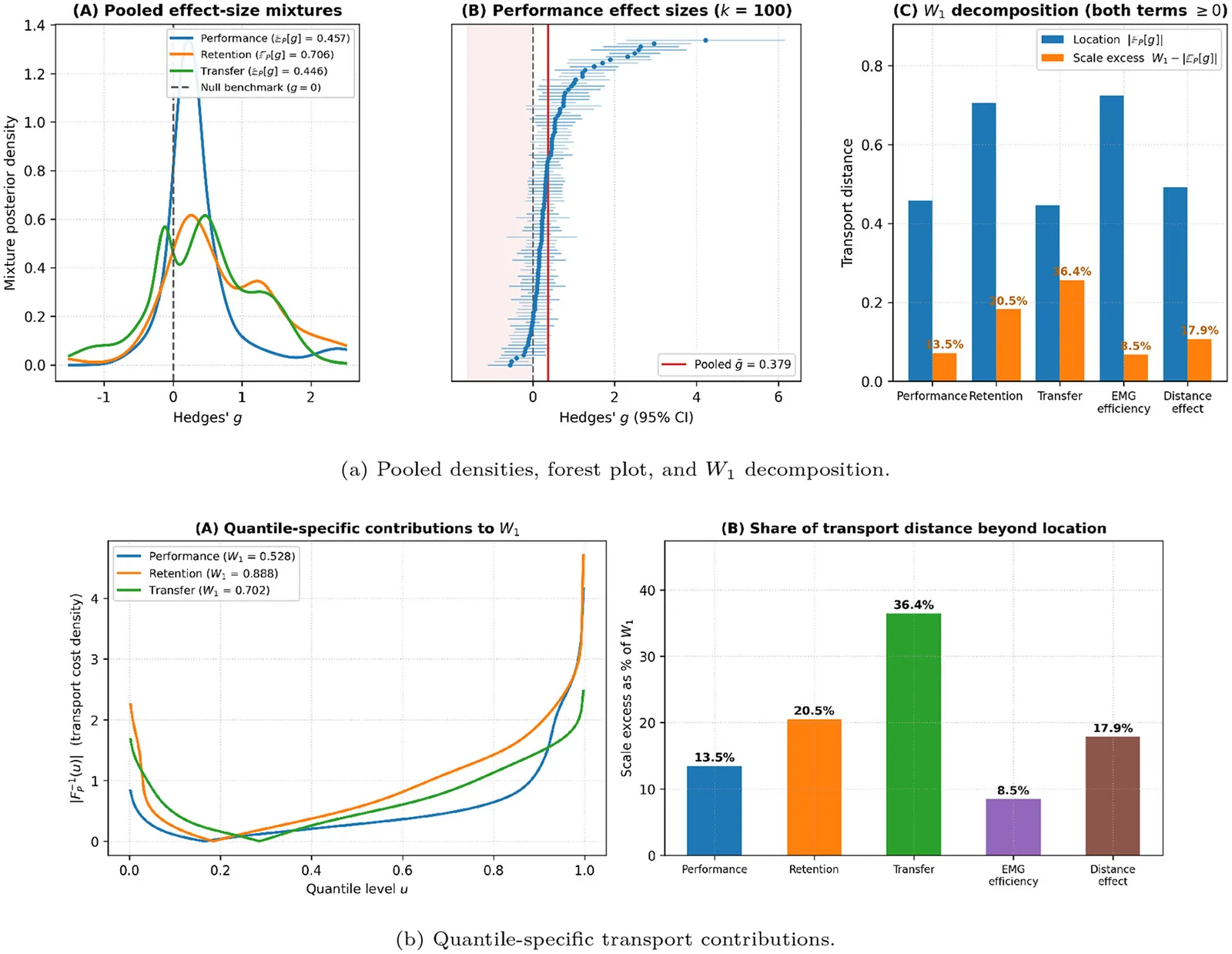
Distributional and quantile-specific views of the attentional-focus meta-analyses. **(a)** A: pooled mixture densities relative to the zero-effect benchmark (*g* = 0), each labeled by its mixture mean 𝔼_*P*_[*g*] rather than by ḡ. B: all 100 performance effect sizes with 95% intervals, sorted by magnitude; the shaded red region marks negative effects. C: the decomposition *W*_1_ = Location+Scale excess for all five outcomes, with the excess share annotated; both components are non-negative by Proposition 1. **(b)** A: $\left|F_{P}^{-1}\left(u\right)\right|$ against quantile level, whose integral is *W*_1_; each curve touches zero where its mixture crosses the null, and the transfer curve does so latest, which gives its left branch the largest area even though retention's is marginally deeper. A: shows the three principal outcomes only. B: scale excess as a percentage of *W*_1_ across outcomes. All values are read from the analysis output file that also generates Table 1.

### 4.3 Wasserstein distances: the mean is only part of the story

Table 1 (left panel) presents the central result. For every outcome, the transport distance from the null exceeds the location term |𝔼_*P*_[*g*]|, as Proposition 1 requires, and the gap between them—the scale excess—is the portion of the evidence that a conventional meta-analysis does not report. Recall from Equation 5 that this residual is twice the expected negative part of the pooled mixture, so it has a direct reading: It measures how much of the study-level evidence points against the recommended instruction, and how far. One point of notation needs care when reading Table 1. The Location column is |𝔼_*P*_[*g*]|, the absolute mean of the sample-size-weighted pooled *mixture*, whereas ḡ is the inverse-variance random-effects estimate. The two answer different questions and need not coincide: For transfer, |𝔼_*P*_[*g*]| = 0.446 sits below ḡ = 0.506 because the random-effects estimator upweights precise studies, while for performance it sits above (0.457 versus 0.379) because several large-sample studies report large effects. We report both rather than relabel either, and the decomposition in Equation 4 is internally consistent throughout: Location + Scale excess = *W*_1_ in every row.

**Table 1:** Distributional and heterogeneity summaries for the five outcomes.

	Wasserstein summaries (benchmark: *g* = 0)						Heterogeneity comparison				
Outcome	*k*	ḡ	*W*_1_	Location	Scale excess	% Excess	*I* ^2^	1−*I*^2^/100	*H_W_*	95% CI for *H_W_*	Divergence
Performance	100	0.379	0.528	0.457	0.071	13.5%	76.3%	0.24	0.47	[0.36, 0.56]	+0.24
Retention	49	0.680	0.888	0.706	0.182	20.5%	79.7%	0.20	0.29	[0.17, 0.42]	+0.09
Transfer	23	0.506	0.702	0.446	0.256	36.4%	83.5%	0.17	0.31	[0.16, 0.50]	+0.14
EMG efficiency	16	0.751	0.792	0.725	0.067	8.5%	87.8%	0.12	0.25	[0.02, 0.50]	+0.13
Distance effect	12	0.318	0.598	0.491	0.107	17.9%	77.6%	0.22	0.66	[0.18, 0.83]	+0.44

(Left) Wasserstein summaries of the pooled effect-size posterior mixtures relative to the zero-effect benchmark: *k* = number of independent effect sizes; ḡ = random-effects pooled Hedges' *g*; *W*_1_ = 𝔼_*P*_|*g*| = total transport distance; Location = |𝔼_*P*_[*g*]|; Scale excess $=W_{1}-\text{Location}=2{𝔼}_{P}\left[g^{-}\right]\geq 0$ (Proposition 1); % Excess = scale excess as a share of *W*_1_; CIs are bootstrap 95% intervals over *B* = 2, 000 study resamples. (Right) Heterogeneity comparison: *I*^2^ = conventional heterogeneity index; 1−*I*^2^/100 = the location-only reference value against which *H_W_* is compared; *H_W_* = distributional heterogeneity; Divergence = *H_W_* − (1 − *I*^2^/100), with positive values indicating shape agreement exceeding what location heterogeneity alone would predict.

Bootstrap 95% intervals for *W*_1_ are [0.399, 0.687] (performance), [0.693, 1.100] (retention), [0.539, 0.905] (transfer), [0.489, 1.162] (EMG efficiency), and [0.268, 1.307] (distance effect); the corresponding intervals for the scale excess are [0.043, 0.109], [0.057, 0.421], [0.061, 0.532], [0.015, 0.126], and [0.018, 0.267]. Every scale-excess interval excludes zero, so in none of the five literature studies is the pooled mean a complete summary of the study-level evidence. We state plainly what that exclusion does and does not establish. Because the study posteriors are Gaussian, each places some mass below zero, so a strictly positive excess follows from the model rather than from the data; the intervals are informative about the *magnitude* of the excess, not about whether it is non-zero. The lower interval limits—0.043 for performance, 0.057 for retention, 0.061 for transfer—are what carry the substantive content because they place the residual at a non-trivial fraction of the transport distance even at the pessimistic end of the resampling distribution.

Transfer shows the largest departure from a mean-only summary. Its transport distance is *W*_1_ = 0.702 against a location term of 0.446, so the scale excess of 0.256 accounts for 36.4% of the total. By Equation 5, this says that the transfer literature places substantial probability mass below zero: Pooling the study posteriors, the expected negative part is 0.128, which is a quarter of the random-effects estimate ḡ = 0.506. The transfer recommendation is therefore the one whose supporting evidence is least well captured by its average. Retention is intermediate (*W*_1_ = 0.888, excess 0.182, 20.5%), and performance—despite being the largest and most frequently cited literature—sits lower (*W*_1_ = 0.528, excess 0.071, 13.5%).

The two smaller meta-analyses bracket this range. The distance effect has an excess share of 17.9%, close to retention. EMG efficiency has the smallest share of all (8.5%; excess 0.067 on *W*_1_ = 0.792), even though its *I*^2^ is the highest in the table at 87.8%.

That last pairing is the most instructive result in Table 1, and it is worth unpacking because the reason is not the obvious one. EMG efficiency has the *highest* *I*^2^ and the *lowest* scale excess; transfer has a lower *I*^2^ (83.5%) and by far the highest excess. It would be natural to assume that EMG's small excess means little of its evidence points the wrong way, but that is not so: The EMG mixture places 20.2% of its mass below zero, *more* than retention (18.5%) or performance (16.5%).

The identity in Equation 5 explains the discrepancy. The excess is $2{𝔼}_{P}\left[g^{-}\right]$, which weights negative mass by how far below zero it falls, so it is a product of frequency and depth rather than a count. Conditional on being negative, the EMG mixture sits only 0.167 below zero, against 0.448 for transfer and 0.494 for retention. EMG's contrary evidence is common but shallow—many studies with intervals that just cross zero—whereas transfer's is both more frequent and roughly three times deeper. That distinction is invisible to *I*^2^, which registers only that the study means are dispersed, and it is equally invisible to any measure of how often the evidence turns negative. The two such measures available here disagree with each other and neither tracks the excess: The share of the pooled mixture below zero ranks EMG above performance (20.2% against 16.5%), while a raw count of negative effect sizes ranks it below (2 of 16 against 14 of 100). It is the kind of structure the transport decomposition is for.

Figure 2a makes this concrete. Panel A shows the pooled mixture densities relative to the zero-effect benchmark, where the leftward mass of the transfer and retention mixtures is directly visible. Panel B presents the forest plot for all 100 performance effect sizes sorted by magnitude, with the shaded red region marking negative effects (internal focus > external focus). Panel C plots the decomposition of Equation 4 for all five outcomes; both bars are non-negative by Proposition 1, and the annotated percentages are the excess shares from Table 1.

Figure 2b offers a finer-grained view. Panel A plots $\left|F_{P}^{-1}\left(u\right)\right|$ against the quantile level *u*, so that the area under each curve is exactly *W*_1_ and the shape of the curve shows *where* in the pooled distribution the transport cost accumulates. Each curve touches zero at the quantile where the mixture crosses the null, so the width of the region to its left is the frequency of contrary evidence and the height of the curve within that region is its depth. The transfer mixture crosses at *u* = 0.286, against 0.185 for retention and 0.165 for performance. Its left branch is not the deepest—averaged over that region, retention reaches 0.494 against transfer's 0.448—but it is much the widest, and it is the area, not the height, that enters the excess. Both factors enter the excess, which is why transfer leads on it while EMG efficiency—whose crossing point is 0.202 but whose left branch is shallow—does not. Panel B summarizes the excess shares across all five outcomes. These are features of the study-level effect-size posterior mixtures, not reconstructions of participant-level external-focus and internal-focus outcome distributions; the distinction is developed in Section 5.5.

### 4.4 Distributional heterogeneity: disagreement about shape, not just size

Table 1 (right panel) compares *H_W_* with *I*^2^ across all five meta-analyses. The two quantities coincide when studies disagree exclusively about location; when they also disagree about shape (variance, skewness, or tail weight), *H_W_* separates from 1−*I*^2^/100, and the size of the separation measures the additional agreement that a variance-based index cannot see.

Every outcome lies above the location-only prediction, and by a wide margin. For performance, *H_W_* = 0.47 against 1−*I*^2^/100 = 0.24, a divergence of +0.24: the conventional index, reading *I*^2^ = 76.3%, would predict that a single summary shape represents barely a quarter of the evidence, whereas the Wasserstein barycenter in fact absorbs almost half of the transport cost. The distance effect shows the largest divergence in the table (*H_W_* = 0.66 versus 0.22, divergence +0.44): Despite *I*^2^ = 77.6%, its twelve studies share a common distributional form closely enough that a single barycentric shape absorbs two-thirds of the transport cost to the naive pool.

At the other end, retention (*H_W_* = 0.29), transfer (*H_W_* = 0.31), and EMG efficiency (*H_W_* = 0.25) show genuinely low shape agreement in absolute terms, even though each still exceeds its location-only prediction. For these three literature studies the barycenter reduces transport cost by only a quarter to a third, meaning that no single distributional shape adequately represents the constituent studies: Some report large, tightly estimated effects, others small and diffuse ones. A pooled estimate such as the retention ḡ = 0.680 is a poor stand-in for studies at either extreme.

The bootstrap intervals qualify how firmly these values are established. Performance, with *k* = 100, has the tightest interval ([0.36, 0.56]) and is comfortably above its 1−*I*^2^/100 benchmark of 0.24. EMG efficiency (*k* = 16, CI [0.02, 0.50]) and the distance effect (*k* = 12, CI [0.18, 0.83]) have intervals wide enough that their point estimates should be treated as indicative rather than precise; with a dozen studies, shape agreement simply cannot be pinned down. We therefore rest the substantive argument on performance, retention, and transfer and report the two smaller meta-analyses for completeness.

A natural question is whether *H_W_* adds information beyond what a conventional prediction interval already conveys (Kepes et al., 2024). The 95% prediction intervals reported in Section 4.1 are wide and zero-spanning for every outcome, and they are wide in much the same way: Performance [−0.33, 1.09] and EMG efficiency [−0.52, 2.02] both tell a practitioner that a new study could land on either side of zero. However, the two differ in *H_W_* (0.47 versus 0.25) and in scale excess (13.5% versus 8.5%). A prediction interval quantifies how far true study-level means may spread; it cannot distinguish a literature whose studies disagree only about magnitude from one whose studies disagree about distributional form, nor tell a reader how much of the pooled evidence actually falls on the wrong side of the null. The transport summaries supply both distinctions, which is why we report them alongside, rather than instead of, τ^2^, *I*^2^, and prediction intervals. We also note the boundary behavior explicitly: When study posteriors are Gaussian with equal variance, transport to the barycenter is purely locational and *H_W_* = 1 exactly when *I*^2^ = 0 (Proposition 2). The two indices therefore agree at the homogeneous boundary, and the divergences reported above arise only when studies differ in posterior width as well as in location.

Figure 3 visualizes the *H_W_*–*I*^2^ relationship and the DRO analysis side by side: Panel A plots each outcome in the *H_W_*–*I*^2^ plane with bootstrap intervals, where points above the dashed line indicate shape agreement exceeding the location-only prediction, and Panel B presents the DRO results developed next.

**Figure 3:**
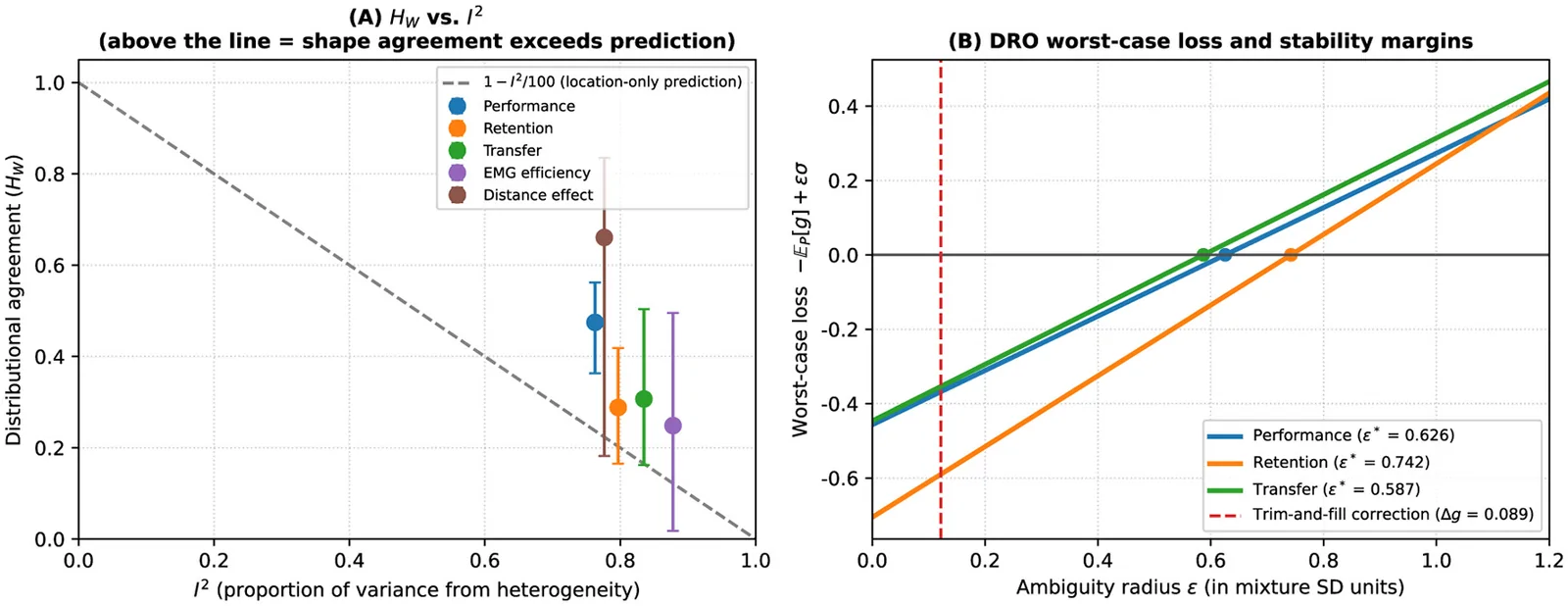
**(A)** *H_W_* vs. *I*^2^ with bootstrap 95% intervals: Points above the dashed line indicate that studies agree more on distributional shape than variance alone would predict. All five outcomes lie above it. **(B)** Worst-case loss −𝔼_*P*_[*g*]+εσ_*P*_ as a function of the ambiguity radius ε, measured in mixture standard deviations. Each curve crosses zero at its stability margin ε^*^, Equation 18: 0.626 for performance, 0.742 for retention, and 0.587 for transfer. The vertical dashed line marks the trim-and-fill correction for performance expressed in the same units (Δ*g* = 0.089, i.e., 0.12σ), which every margin exceeds comfortably.

### 4.5 Stability of the external-focus recommendation

The DRO analysis, introduced in Section 3.5, asks how much distributional perturbation the external-focus recommendation can withstand before it ceases to be optimal. By Equation 18, the stability margin is $\varepsilon^{*}={𝔼}_{P}\left[g\right]/\sigma_{P}$, expressed in mixture standard deviations. We use “stability margin” rather than “robustness margin” to avoid confusion with the statistical sense of robustness: ε^*^ is a worst-case threshold, not a claim about estimator properties.

The margins are ε^*^ = 0.626 for performance, 0.742 for retention, 0.587 for transfer, 0.949 for EMG efficiency, and 0.411 for the distance effect. To compare these against publication-bias corrections, the corrections must be converted to the same units. The trim-and-fill correction we compute for performance is Δ*g* = 0.089 on a mixture standard deviation of σ_*P*_ = 0.730, or 0.12σ; for retention, Δ*g* = 0.197 on σ_*P*_ = 0.951, or 0.21σ; for transfer, Δ*g* = 0.323 on σ_*P*_ = 0.761, or 0.42σ. Each stability margin exceeds its corresponding correction, so on this evidence the external-focus recommendation survives trim-and-fill adjustment in all three principal outcomes.

The ordering, however, is not the one the pooled means suggest. Transfer has a mid-sized pooled effect but the narrowest margin relative to its own correction (0.587 against 0.42σ) because its mixture is both widely spread and asymmetric. EMG efficiency has the largest margin of all (0.949) despite resting on only 16 studies because its evidence is unusually one-directional. The distance effect has the smallest margin (0.411) on the widest mixture (σ_*P*_ = 1.197), driven by a single extreme estimate; with *k* = 12 this margin should be regarded as unstable.

Two qualifications bound the interpretation. First, ε^*^ is defined relative to the pooled mixture posterior, not to a comparison of external-focus and internal-focus participant-level outcome distributions; it quantifies the stability of the study-level evidence, not the worst-case outcome for an individual participant. Second, as Section 3.5 states, in this univariate setting ε^*^ is a signal-to-noise ratio, and its ordering across outcomes therefore reflects the width of each mixture as much as the size of each effect. The value of the DRO framing is that it places effects and bias corrections on a common scale, not that it introduces evidence beyond what 𝔼_*P*_[*g*] and σ_*P*_ already contain.

### 4.6 Publication bias: shape distortion vs. mean shift

Table 2 reports three publication-bias diagnostics computed on the same corpus: the Egger et al. (1997) regression-intercept test, the Duval and Tweedie (2000) trim-and-fill correction, and the KL divergence between the published and filled mixtures. Because we compute the trim-and-fill correction ourselves rather than quoting a published figure, the mean shift and the shape comparison refer to the same imputation and are directly comparable.

**Table 2:** Publication-bias diagnostics computed on the full corpus.

Outcome	Egger intercept	Egger *p*	*k*_0_	Trim-fill |*Δg*|	*D*_KL_
Performance	2.05	<0.001	2	0.089	0.020
Retention	2.95	0.006	3	0.197	0.031
Transfer	3.06	0.007	3	0.323	0.055
EMG efficiency	4.39	0.032	2	0.291	0.090
Distance effect	2.95	0.074	1	0.107	0.080

Egger intercept and *p* from the regression asymmetry test; *k*_0_ = number of studies imputed by trim and fill; |Δ*g*| = resulting shift in the pooled estimate; *D*_KL_ = Kullback–Leibler divergence between the published and filled mixtures, a shape comparison that is insensitive to the size of the mean shift.

The asymmetry evidence is considerably stronger here than in the smaller corpus analyzed by Chua et al. (2021). Egger's test is significant for four of the five outcomes, and for performance it is decisively so (intercept = 2.05, *p* < 0.001), where the earlier synthesis had reported a non-significant result. On the larger corpus, in other words, the funnel asymmetry that McKay et al. (2024) emphasized is clearly present.

What the KL column adds is a statement about *kind* rather than degree. The divergences are small in absolute terms—0.020 for performance, rising to 0.090 for EMG efficiency—which means the published and filled mixtures remain close in overall shape even where the mean shift is substantial. Transfer illustrates the dissociation most clearly: Its trim-and-fill correction is the largest in the table (|Δ*g*| = 0.323, a 64% reduction in the pooled estimate), yet its shape divergence is middling (*D*_KL_ = 0.055). Imputing three studies moves the center of the transfer distribution a long way without greatly changing its form. Conversely, EMG efficiency has a smaller mean shift but the largest shape divergence because imputing two studies into a corpus of sixteen alters the mixture's structure disproportionately.

The practical reading is that mean-shift and shape-distortion diagnostics answer different questions and can disagree. An analyst who asks only “how much does the pooled estimate move?” would rank transfer as the most bias-affected outcome; one who asks “how differently is the evidence distributed?” would rank EMG efficiency first. We report both because the choice between publication-bias corrections in this literature has turned on precisely this ambiguity. Figure 4 shows the performance funnel plot alongside the two diagnostics across all five outcomes.

**Figure 4:**
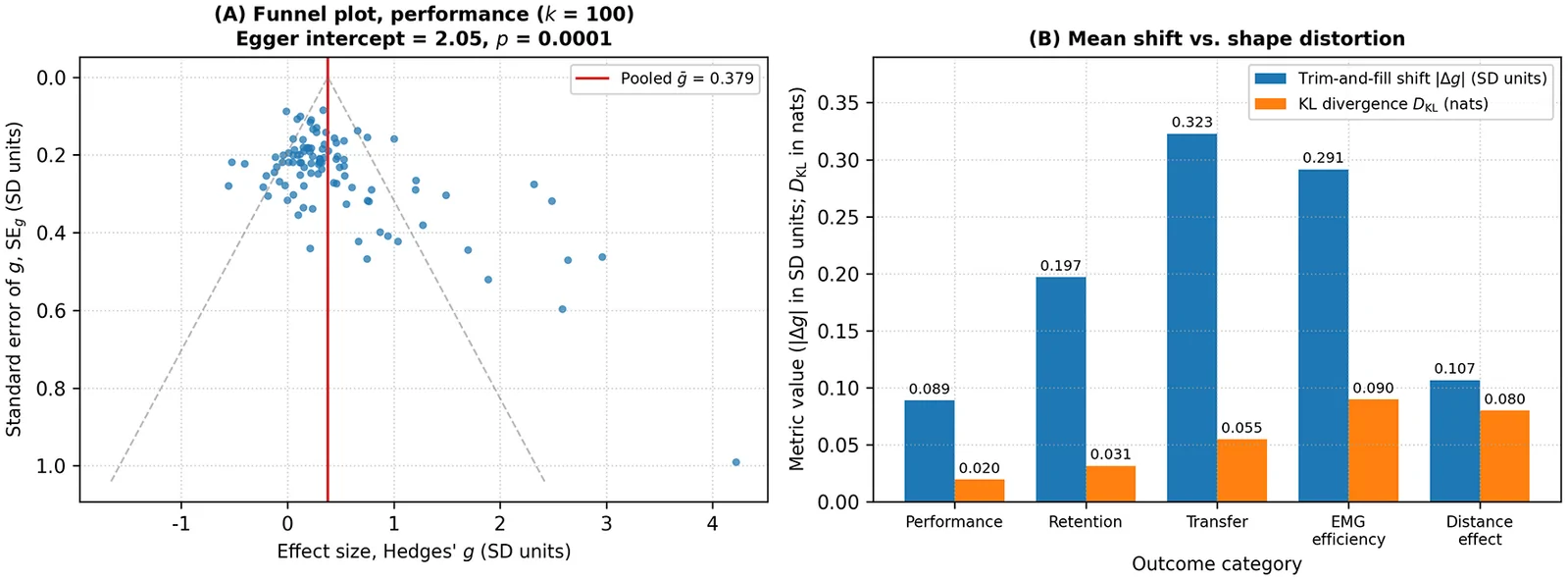
Publication-bias diagnostics. **(A)** Funnel plot of all 100 performance estimates, with the pooled mean and pseudo-confidence contours; the Egger intercept and *p*-value are annotated. **(B)** Trim-and-fill mean shift |Δ*g*| and the KL divergence between published and filled mixtures, plotted side by side for all five outcomes. The two diagnostics do not rank the outcomes alike: Transfer has the largest mean shift but a middling shape divergence, while EMG efficiency has the largest shape divergence on a smaller shift.

### 4.7 Sensitivity analysis

We assess four analytical choices. The first three leave the substantive conclusions intact; the fourth identifies a genuine limitation, which we report rather than suppress.

#### 4.7.1 Posterior width

Doubling the variance of every study-level posterior (using $2s_{i}^{2}$) increases *W*_1_ by 3.4% to 5.6% across outcomes—from 0.528 to 0.558 for performance, 0.888 to 0.932 for retention, and 0.702 to 0.734 for transfer—and lowers *H_W_* by at most 0.023. Wider posteriors push more mass across zero and so must increase the scale excess; the fact that a 100% change in assumed variance moves *W*_1_ by under 6% indicates the transport summaries are not artifacts of posterior specification.

#### 4.7.2 Standard-error formula

Substituting the truncated shortcut $s_{i}\approx \sqrt{4/n_{i}}$ for the exact standard errors changes *W*_1_ by +18.0% for performance (0.528 to 0.623), +10.8% for EMG efficiency, and +13.8% for the distance effect, while retention and transfer move by under 3%. The effect on *H_W_* is far larger: It falls from 0.47 to 0.21 for performance and from 0.25 to 0.04 for EMG efficiency. This is a substantial distortion, and it runs in the direction the algebra predicts—the shortcut understates the uncertainty of large-effect studies, which sharpens their posteriors and makes them appear to disagree more with the consensus shape than they do. The magnitude of the distortion is itself a finding: Distributional summaries are considerably more sensitive to standard-error approximation than pooled means are, so a reanalysis of this kind requires exact standard errors rather than sample-size proxies.

#### 4.7.3 Outlier influence

Removing the three largest and three smallest effect sizes changes ḡ by −4.2% and *W*_1_ by −12.6% for performance and by −4.6% and −15.0% for retention. Contrary to what we might have expected, the transport summary is *more* sensitive to trimming than the mean, not less: Because the scale excess is driven by mass below zero, deleting the most negative studies removes precisely the observations that generate it. Readers should therefore treat the scale excess as a statistic about the tails of the evidence, with the fragility that implies.

#### 4.7.4 Mixture weighting

Replacing sample-size weights with equal weights raises *W*_1_ by 6.0% to 10.7% for the three principal outcomes, leaving the ordering unchanged. For the distance effect, however, it raises *W*_1_ by 35.9% (0.598 to 0.813), and trimming six studies lowers it by 54.6%. With *k* = 12 and one estimate as large as *g* = 5.25, that meta-analysis is too small for its transport summaries to be stable under any weighting scheme. We report its values in Table 1 for completeness but exclude the distance effect from the substantive claims of Section 5. EMG efficiency (*k* = 16) needs a narrower qualification. Its *H_W_* is unstable—it falls from 0.25 to 0.04 under the truncated standard error—and we treat that value as indicative only, consistent with Section 4.4. Its scale excess, by contrast, is the smallest in the corpus under every variant we ran (equal weights 6.0%, trimming 2.3%, doubled posterior width 11.5%, truncated standard error 17.4%), so the EMG–transfer contrast on which we rely elsewhere does not depend on the weighting or posterior-width choices.

## 5 Discussion

This study began with a simple observation: Meta-analyses of attentional focus have mostly compared average effects, leaving the broader shapes of the study-level effect-size distributions relatively unexamined. Our reanalysis of the McKay et al. (2024) corpus using three distributional tools suggests that the external-focus literature contains information beyond the pooled mean. Across five meta-analyses, the Wasserstein-1 distance (*W*_1_) identifies a residual—the pooled mass lying against the recommendation, weighted by its depth—that Hedges' *g* cannot report; the distributional heterogeneity measure (*H_W_*) reveals shape disagreement between studies that *I*^2^ alone obscures; and distributionally robust optimization (DRO) shows that in all three principal outcomes the recommendation withstands perturbations larger than the publication-bias corrections we compute. We discuss these results in terms of their bearing on theory, practice, and meta-analytic methodology, while noting that they describe study-level effect-size mixtures rather than participant-level outcome distributions.

### 5.1 Theoretical implications

Wulf and Lewthwaite (2016) proposed the OPTIMAL theory of motor learning, which identifies external focus as one of several conditions that promote automaticity. The scale excess we document for transfer (0.256, or 36.4% of its transport distance) and retention (0.182, 20.5%) indicates that the pooled study-level evidence is not exhausted by the mean alone, although the interpretation is narrower than what raw-outcome data would permit. Constrained-action theory, as articulated by Lohse et al. (2010) and Lohse et al. (2014), explains the mean shift as a product of more automatic motor control under external focus. The present findings motivate future work examining whether the same mechanism also predicts differences in variability and tails at the participant level, but the current data do not directly identify those within-condition outcome distributions.

Second, the *H_W_* analysis refines what we mean by “heterogeneity” in meta-analysis. Higgins and Thompson (2002) introduced *I*^2^ as a measure of between-study variance relative to total variance, and it has become the standard for assessing whether a pooled estimate is representative. However, *I*^2^ captures only location disagreement. Borenstein et al. (2009) acknowledged that heterogeneity is more complex than a single number can convey, and Cumming (2012) warned that averaging across heterogeneous studies yields a pooled estimate that may represent none of them. In our corpus, *H_W_* exceeds 1−*I*^2^/100 for every outcome, and the gap does not track *I*^2^: for the distance effect, *I*^2^ = 77.6% —among the lowest in the corpus—predicts shape agreement of 0.22, but the barycenter in fact absorbs 0.66 of the transport cost. Read through *I*^2^ alone, these literature studies look more disordered than they are.

The sharper lesson concerns what the two indices measure. EMG efficiency has the highest *I*^2^ in the corpus and the smallest scale excess; transfer has a lower *I*^2^ and the largest excess. *I*^2^ is a variance ratio, sensitive to precise studies that disagree about magnitude. The scale excess is $2{𝔼}_{P}\left[g^{-}\right]$: It weights contrary evidence by how far it falls below zero, so it responds to the frequency *and* the depth of disagreement about direction. The EMG and transfer literature studies both place substantial mass below zero (20.2% and 28.6%), but conditional on being negative the EMG mixture sits only 0.167 below the null against transfer's 0.448. A practitioner told that both literature studies are “highly heterogeneous” learns nothing about that difference, and a practitioner told only the pooled means learns less still. The right question for moderator analysis is correspondingly not only “which subgroups have larger effects?” but “which subgroups place more evidence against the recommendation?”—a richer form of moderation than the tradition of *Q*-tests and subgroup analysis allows (Hedges, 1981; DerSimonian and Laird, 1986).

### 5.2 Resolving the Chua–McKay debate

The tension between Chua et al. (2021) and McKay et al. (2024) has dominated recent discussion in the attentional-focus literature. Chua et al. (2021) concluded that external focus produces a statistically significant mean advantage (*g* = 0.264 for performance); McKay et al. (2024) counter-argued that publication-bias correction deflates this effect substantially. Our results speak to this exchange in two ways, one supporting each side.

On the side of caution, we find clear funnel asymmetry in the larger corpus. Egger's test is significant for four of five outcomes and decisively so for performance (*p* < 0.001), where the smaller corpus had not shown it. On the evidence assembled here, the concern that motivated the Bayesian re-analysis is well founded.

On the other side, the shape diagnostics suggest that asymmetry and distortion are not interchangeable. The KL divergences between published and filled mixtures are small throughout (0.020–0.090), and—more tellingly—they do not track the mean shifts. Transfer sustains the largest trim-and-fill correction in the corpus, a 64% reduction in its pooled estimate, on a middling shape divergence; EMG efficiency shows the opposite pattern. A correction can move the center of a literature a long way while leaving its form largely intact, and a debate conducted only in terms of corrected means will not register that distinction. We therefore read the Chua–McKay disagreement as partly a disagreement about which question to ask of the same data, rather than solely about the value of a parameter.

The DRO analysis adds a scale on which the two can be compared. Expressed as signal-to-noise ratios, the stability margins (ε^*^ = 0.626, 0.742, and 0.587 for performance, retention, and transfer) each exceed the corresponding trim-and-fill correction in the same units (0.12σ, 0.21σ, and 0.42σ). Transfer has the narrowest relative margin, which is consistent with its being the outcome whose evidence is least concentrated on one side of the null. Two limits bound this reading: ε^*^ is computed relative to a point-mass null and pertains to the pooled study-level posterior, so it is not a test of the external-versus-internal comparison at the participant level; and, as Section 3.5 notes, in the univariate case ε^*^ contains no information beyond 𝔼_*P*_[*g*] and σ_*P*_. Its contribution is commensurability, not additional evidence.

### 5.3 Practical implications

The distributional findings have practical bearing because many real-world decisions depend on the spread of evidence, not just its average. In clinical rehabilitation, a fall or a re-injury is qualitatively different from a mediocre training session; practitioners care about how stable the evidence is, not only what its mean says. Our analysis suggests that the retention and transfer literature studies contain structured distributional information beyond the pooled mean. In rehabilitation settings, where an adverse event carries consequences a training study does not capture, that observation motivates future participant-level work on risk and tails. The present study, however, should be read as evidence about the shape of study-level effects rather than direct evidence about the frequency of catastrophic patient outcomes.

In competitive sport, the question reverses: Coaches and athletes care about the right tail—the probability of exceptional performance. Our current analysis, constrained to study-level posteriors, cannot directly quantify right-tail differences. Individual-participant data would allow decomposition of right-tail contributions to *W*_1_; until such data are available, we can only say that the study-level evidence for external focus is not summarized fully by the mean alone. Wulf et al. (2001) argued that external focus enhances movement automaticity; the present results motivate, but do not by themselves establish, the claim that such automaticity yields more predictable upper-end performance.

In physical education and group instruction, the relevant question is what works for the most people, including the weakest performers. A distribution with fewer extreme negative outcomes is safer when instructors cannot monitor each individual. The *H_W_* analysis adds a caution: When *H_W_* is low, as it is for retention (0.29), transfer (0.31), and EMG efficiency (0.25), the average effect represents its constituent studies poorly, and practitioners should consider whether their specific domain resembles the studies that found large, consistent effects or those that found small, variable ones.

### 5.4 Methodological contributions

Beyond attentional focus, our approach offers three methodological contributions. The Wasserstein-1 distance with quantile decomposition provides a natural metric for comparing meta-analytic distributions. Unlike Cohen's *d* or Hedges' *g*, which compare only the means, *W*_1_ integrates differences across quantiles and can reveal distributional structure that a pooled mean misses (Panaretos and Zemel, 2019). Unlike the Kullback-Leibler divergence, *W*_1_ is symmetric, defined for distributions with non-overlapping support, and has a clear geometric interpretation as the cost of transforming one distribution into another (Villani, 2009; Peyré and Cuturi, 2019). The heterogeneity measure *H_W_* complements *I*^2^ by capturing distributional similarity rather than just mean similarity; when they diverge, *H_W_* signals that studies disagree in form, not merely in magnitude. Distributionally robust optimization, drawn from recent work in operations research (Kuhn et al., 2019), reframes the question “is the effect real?” as “how much perturbation can the recommendation withstand?”—a formulation that is directly relevant to meta-analytic inference under publication bias.

These contributions should be read as applications rather than new statistical theory: The transport metrics, the barycenter construction, and the DRO machinery are taken directly from Panaretos and Zemel (2019), Cuturi and Doucet (2014), and Kuhn et al. (2019), and the replication-oriented distributional perspective from Mathur and VanderWeele (2020). They also sit within a broader movement to take heterogeneity assessment beyond *I*^2^. Kepes et al. (2024) documented how rarely meta-analyses interpret heterogeneity statistics thoroughly and recommended routine prediction-interval reporting; Holzmeister et al. (2024) partitioned heterogeneity into population, design, and analytical components; and Krefeld-Schwalb et al. (2025) showed how the sampled populations constrain what population heterogeneity can even be estimated. Those frameworks ask where the variation in effect sizes comes from; the transport decomposition asks what the pooled evidence looks like once the variation is present. The two questions are complementary, and syntheses that report τ^2^, prediction intervals, and a shape-sensitive index such as *H_W_* together will convey more than any single summary alone.

### 5.5 Limitations and future directions

The analysis carries several limitations. The study-level posteriors are Gaussian ($P_{i}=N\left(g_{i},s_{i}^{2}\right)$), an assumption that may not hold for studies with small samples or skewed outcomes. More resilient bootstrap-based or Bayesian posterior-predictive distributions would require raw participant-level data. The source dataset provides only study-level summary statistics, so within-study distributional analyses were not possible, and the present study cannot directly identify participant-level external-focus and internal-focus outcome distributions. The DRO analysis is also one-dimensional (effect sizes only), whereas motor performance is multidimensional; extending Wasserstein methods to multivariate outcome spaces is a natural but complex next step (Cuturi and Doucet, 2014; Galichon, 2016). Finally, a caveat raised by the agreement between *H_W_* and *I*^2^ at the equal-variance Gaussian boundary (Proposition 2): Transport summaries computed from study-level Gaussian posteriors are necessarily related to variance-based heterogeneity, and although the two indices demonstrably diverge in this corpus, that divergence is driven by differences in posterior width rather than by any structure the summary statistics do not already contain. Without participant-level data, the shape differences we report cannot be validated against ground-truth outcome distributions, and they should be read as descriptive summaries of the study-level evidence.

Three extensions follow from this study. Participant-level data would permit direct right-tail analysis and would show whether external focus also raises the probability of exceptional performance rather than only the average effect. Meta-analytic prediction intervals (Cumming, 2014) already quantify the range of true effects, and combining them with *H_W_* would help separate “wide prediction interval because studies disagree on the mean” from “wide prediction interval because studies disagree on the shape.” The Wasserstein framework also extracts additional information from the same summary statistics that conventional meta-analysis already uses, which makes it immediately applicable to the many existing syntheses for which only effect sizes and standard errors are available.

## 6 Conclusion

The question “Does external focus work better than internal focus?” has usually been answered with a mean. Our reanalysis of 200 effect sizes shows that the answer is incomplete in a way that can be measured rather than merely asserted. Against a zero-effect benchmark, the transport distance splits exactly into the pooled mean and a non-negative residual equal to twice the expected negative part of the evidence—the mass pointing against the recommendation. That residual is 36.4% of the transport distance for transfer, 20.5% for retention, and 13.5% for immediate performance, and its bootstrap interval excludes zero in every outcome we examined.

The residual is not a relabeling of heterogeneity. The outcome with the highest *I*^2^ in the corpus, EMG efficiency at 87.8%, has the smallest residual at 8.5%—not because little of its evidence points the wrong way, but because what does lies shallowly, just below the null. Transfer's contrary evidence is both more frequent and roughly three times deeper. Because the residual weights contrary mass by its depth, it separates these cases; *I*^2^ does not, and a count of negative studies registers only how often the evidence turns, never how far.

For coaches, therapists, and educators, the practical message is therefore not the bare claim that “external focus is better.” Every pooled estimate in this corpus is positive, but every prediction interval includes zero, and the transfer literature in particular carries substantial evidence pointing the other way. For meta-analysts, the message is that a single additional number—the expected mass on the wrong side of the null, available from the same summary statistics that conventional syntheses already use—conveys something that neither the pooled mean nor *I*^2^ reports. Stronger claims about participant-level tails, catastrophic failures, or within-condition variance will require richer data than the study-level summaries available to us.

## Data Availability

The original contributions presented in the study are included in the article/Supplementary material, further inquiries can be directed to the corresponding author.
