# Supplementary material for: Beyond the pooled mean: distributional structure in the attentional-focus meta-analysis
Source: Front Psychol. 2026 Sep 28;17:1916455. doi: 10.3389/fpsyg.2026.1916455 (PMC13655098; doi:10.3389/fpsyg.2026.1916455)
Supplement: Supplementary file 1 [file Supplementary_file_1.docx]

**Supplementary Material: Study-Level Data for
“Beyond the Pooled Mean: Distributional Structure in the Attentional-Focus Meta-Analysis”**

**Yibo Zeng**1, **Xupeng Zhang**2,*, **Pan Wang**1
*1 College of Physical Education, Xihua University, Chengdu, Sichuan 610039, China
2 Sichuan Vocational and Technical College of Communications, Chengdu 611130, China
* Corresponding author: zhangxupeng@svtcc.edu.cn*

# Overview and Description of Study-Level Records

This supplementary document provides the complete study-level records for all 200 effect sizes analysed in the main manuscript. The dataset was compiled and distributed by McKay et al. (2024), which updated and extended the earlier systematic review and meta-analysis of Chua et al. (2021). All included studies compared external-focus and internal-focus instructions in motor tasks, using Hedges' g as the standardized effect size metric (positive values denote an external-focus advantage).

The 200 effect sizes are grouped across five motor outcome categories:
• Table S1: Immediate performance (k = 100 effect sizes, N = 2,182)
• Table S2: Retention (k = 49 effect sizes, N = 1,614)
• Table S3: Transfer (k = 23 effect sizes, N = 797)
• Table S4: EMG efficiency (k = 16 effect sizes, N = 299)
• Table S5: Distance effect (k = 12 effect sizes, N = 287)

**Column Definitions:**
• Study: Primary study identifier (first author and year / experiment number).
• n: Total sample size across intervention and control groups.
• g: Hedges' g (standardized mean difference; positive values favor external focus).
• s: Exact standard error (SE_g) as reported in the source dataset.
• Task: Specific motor task investigated.
• Risk of bias: Methodological risk of bias rating (Low, Moderate, or High).

## Table S1: Performance effect sizes (k = 100, N = 2182)

| **Study** | **n** | **g** | **s** | **Task** | **Risk of bias** |
| --- | --- | --- | --- | --- | --- |
| Abdollahipour & Psotta (2017) | 24 | 0.459 | 0.211 | Ball catching | Moderate |
| Abdollahipour et al. (2015) | 24 | 0.237 | 0.204 | Jumping | Moderate |
| Abdollahipour et al. (2016) | 24 | 0.097 | 0.201 | Jumping | Low |
| Asadi et al. (2015) | 21 | 0.299 | 0.219 | Javelin throwing | Low |
| Ashraf et al. (2017) | 20 | 0.118 | 0.220 | Jumping | Low |
| Beck & Almeida (2017) | 19 | 0.054 | 0.159 | Balance | Low |
| Coker (2016) | 21 | 0.533 | 0.229 | Jumping | Moderate |
| Coker (2018) | 26 | 0.471 | 0.204 | Jumping | Moderate |
| de Melker Worms et al. (2017) | 28 | 0.066 | 0.187 | walking | Low |
| Diekfuss et al. (2018) | 8 | 0.872 | 0.399 | Balance | Low |
| Ducharme & Wu (2015) | 29 | 0.385 | 0.190 | Balance | Low |
| Ducharme et al. (2015) | 21 | 0.308 | 0.219 | Jumping | Low |
| Duke et al. (2011) | 12 | -0.228 | 0.283 | Piano playing | Low |
| Duke et al. (2011) | 4 | 0.214 | 0.441 | Piano playing | Low |
| Durham et al. (2014) | 21 | -0.010 | 0.088 | Reach-to-grasp and object manipulation | Low |
| Fasoli et al. (2002) | 17 | 0.539 | 0.254 | Reach-to-grasp | Low |
| Fasoli et al. (2002) | 16 | 0.288 | 0.249 | and object manipulation | Low |
| Freudenheim et al. (2010 Exp 1) | 38 | 0.146 | 0.161 | Swimming | Low |
| Freudenheim et al. (2010 Exp 2) | 30 | 0.184 | 0.182 | Swimming | Low |
| Greig & Marchant (2014) | 25 | 0.215 | 0.116 | Elbow flexion | Low |
| Halperin et al. (2017) | 8 | 0.236 | 0.339 | Punching | Low |
| Halperin et al. (2017) | 7 | 0.102 | 0.355 | Punching | Low |
| Halperin, Hughes, et al. (2016) | 28 | 0.240 | 0.134 | and jumping | Low |
| Halperin, Williams, et al. (2016) | 18 | 0.323 | 0.237 | Midthigh pull | Low |
| Hebert & Williams (2017) | 27 | 0.154 | 0.191 | Jumping | Moderate |
| Hill et al. (2017) | 30 | 0.150 | 0.181 | Treadmill running | Low |
| Hosseiny et al. (2014) | 10 | 1.889 | 0.521 | shooting | Low |
| Huang et al. (2014) | 12 | 0.767 | 0.320 | Balance | Low |
| Ille et al. (2013) | 8 | 1.040 | 0.423 | Sprinting | Low |
| Ille et al. (2013) | 8 | 0.945 | 0.409 | Sprinting | Low |
| Jazaeri et al. (2018) | 17 | 1.004 | 0.159 | Balance | Low |
| Jazaeri et al. (2018) | 34 | 0.347 | 0.173 | Balance | Low |
| Kal et al. (2013) | 30 | 0.199 | 0.182 | Cyclic single-leg extension-flexion | Low |
| Kearney (2015) | 18 | -0.101 | 0.231 | Golf putting | Low |
| Klostermann et al. (2014) | 24 | 1.209 | 0.266 | Golf putting | Low |
| Kuhn et al. (2017) | 14 | 0.607 | 0.284 | Finger abduction | Low |
| Kuhn et al. (2018 Exp 1 & 2) | 26 | 0.211 | 0.192 | Finger flexion | Low |
| Landers et al. (2005) | 22 | 0.660 | 0.138 | Balance | Low |
| Lisman & Sadagopan (2013) | 20 | 1.205 | 0.290 | Speech | Low |
| Lohse & Sherwood (2011 Exp 1) | 20 | 0.223 | 0.222 | Wall-sit | Low |
| Lohse & Sherwood (2012 Exp 1 & 2) | 24 | 0.273 | 0.142 | Force production (plantar flexion) | Low |
| Lohse et al. (2010) | 12 | 1.274 | 0.381 | Dart throwing | Low |
| Lohse et al. (2011) | 12 | 2.587 | 0.597 | Force production (plantar flexion) | Low |
| Lohse et al. (2014) | 15 | 0.791 | 0.290 | Dart throwing | Low |
| Makaruk et al. (2013) | 30 | 0.274 | 0.130 | Shot put | Low |
| Makaruk et al. (2015) | 23 | 0.303 | 0.210 | Ergometer cycling | Low |
| Marchant & Greig (2017) | 20 | 0.012 | 0.219 | Force production (knee extension) | Low |
| Marchant et al. (2011) | 40 | 0.752 | 0.155 | Bench press and back squat | Moderate |
| Marchant, Carnegie, et al. (2018) | 26 | 0.362 | 0.142 | Golf putting | Low |
| Marchant, Clough, et al. (2009) | 72 | 0.336 | 0.085 | Dart throwing | Low |
| Marchant, Greig, et al. (2009) | 25 | 2.962 | 0.463 | Elbow flexion | Low |
| Marchant, Griffiths, et al. (2018) | 98 | 0.122 | 0.101 | Jumping | Moderate |
| Maurer & Munzert (2013 Exp 1) | 23 | -0.523 | 0.219 | Basketball free throw | Low |
| Maurer & Munzert (2013 Exp 2) | 14 | -0.554 | 0.280 | Golf putting | Low |
| McNevin et al. (2013) | 12 | 0.118 | 0.199 | Supra-postural manual tracking | Low |
| McNevin et al. (2013) | 12 | 0.532 | 0.212 | Supra-postural manual tracking | Low |
| Mornell & Wulf (2019 Exp 1 & 2) | 41 | 0.532 | 0.164 | Music | Low |
| Nadzalan et al. (2015) | 30 | 2.488 | 0.319 | Bench press | Low |
| Neumann & Piercy (2013) | 21 | -0.402 | 0.223 | Treadmill running | Low |
| Oki et al. (2018) | 12 | 1.699 | 0.445 | throwing | Low |
| Palmer et al. (2017) | 44 | 0.442 | 0.157 | Ball control | Low |
| Pelleck & Passmore (2017) | 11 | -0.113 | 0.206 | Golf putting | Low |
| Pelleck & Passmore (2017) | 13 | 0.011 | 0.195 | Golf putting | Low |
| Perkins-Ceccato, et al. (2003) | 10 | -0.183 | 0.306 | Golf pitch shot | Low |
| Perkins-Ceccato, et al. (2003) | 10 | 0.056 | 0.303 | Golf pitch shot | Low |
| Polskaia et al. (2015) | 20 | 0.052 | 0.219 | Postural control | Low |
| Porter & Anton (2011) | 5 | 0.749 | 0.468 | Manual tracking | Low |
| Porter & Sims (2013) | 9 | 0.000 | 0.317 | Sprinting | Low |
| Porter et al. (2013) | 38 | 0.460 | 0.169 | Jumping | Moderate |
| Porter et al. (2015) | 84 | 0.223 | 0.110 | Sprinting | Low |
| Porter, Nolan, et al. (2010) | 20 | 2.639 | 0.471 | Sprinting | Low |
| Querfurth et al. (2016) | 20 | 0.129 | 0.220 | Dart throwing | Low |
| Richer et al. (2017) | 16 | -0.123 | 0.245 | Balance | Low |
| Rossettini et al. (2017) | 10 | 4.221 | 0.991 | tapping | Low |
| Saemi et al. (2016) | 11 | 1.492 | 0.304 | Basketball free | Low |
| Schutts et al. (2017) | 12 | 0.154 | 0.280 | Snatch | Low |
| Schücker et al. (2009) | 24 | 0.345 | 0.207 | Treadmill running | Low |
| Schücker et al. (2015) | 18 | 0.157 | 0.232 | Ergometer | Low |
| Schücker, Anheier, et al. (2013) | 20 | 0.292 | 0.224 | Treadmill running | Low |
| Schücker, Fleddermann, et al. (2016) | 24 | 0.054 | 0.201 | Ergometer cycling | Low |
| Schücker, Schmeing, et al. (2016) | 30 | 0.329 | 0.185 | Treadmill running | Low |
| Shafizadeh, Platt & Mohammadi (2013) | 12 | 0.753 | 0.318 | Treadmill walking | Moderate |
| Sherwood et al. (2014 Exp 1 & 2) | 48 | 2.320 | 0.276 | Dart throwing | Low |
| Stambaugh (2017) | 15 | 0.120 | 0.252 | Playing of musical instrument | Low |
| Stambaugh (2017) | 15 | -0.199 | 0.254 | Playing of musical instrument | Low |
| Stoate & Wulf (2011) | 30 | 0.224 | 0.183 | Swimming | Low |
| Winkelman et al. (2017 Exp 1 & 2) | 16 | 0.221 | 0.247 | Sprinting | Low |
| Winkelman et al. (2017 Exp 1 & 2) | 13 | -0.075 | 0.269 | Sprinting | Low |
| Wu et al. (2012) | 21 | 0.324 | 0.220 | Jumping | Moderate |
| Wulf & Dufek (2009) | 10 | 0.555 | 0.327 | Jumping | Low |
| Wulf & Su (2007 Exp 2) | 6 | 0.669 | 0.423 | Golf pitch shot | Low |
| Wulf et al. (2008) | 12 | -0.022 | 0.279 | Balance | Low |
| Wulf et al. (2009) | 14 | 0.435 | 0.272 | Balance | Low |
| Wulf, Dufek, et al. (2010) | 8 | 0.150 | 0.336 | Jumping | Low |
| Wulf, Töllner, et al. (2007 Exp 1 & 2) | 42 | 0.092 | 0.108 | Balance | Low |
| Wulf, Zachry, et al. (2007 Exp 1 & 2) | 22 | 0.305 | 0.211 | Jumping | Low |
| Yogev-Seligmann et al. (2017) | 20 | -0.047 | 0.219 | Walking | Low |
| Zachry et al. (2005) | 14 | 0.462 | 0.274 | Basketball free | Moderate |
| Zarghami et al. (2012) | 20 | 0.491 | 0.232 | Discus throwing | Low |
| Ziv et al. (2013) | 24 | -0.039 | 0.201 | Treadmill running | Low |

## Table S2: Retention effect sizes (k = 49, N = 1614)

| **Study** | **n** | **g** | **s** | **Task** | **Risk of bias** |
| --- | --- | --- | --- | --- | --- |
| Abdollahipour et al. (2008) | 76 | 0.449 | 0.225 | Soccer dribbling | Moderate |
| Abdollahipour et al. (2014) | 40 | -0.076 | 0.310 | Dart throwing | Moderate |
| Abdollahipour et al. (2019 Exp 2) | 26 | 0.379 | 0.383 | Pedalo riding | Moderate |
| Agar et al. (2016) | 48 | -0.099 | 0.280 | Puck shooting | Low |
| Ahmad et al. (2013) | 44 | 2.622 | 0.407 | Volleyball serve | Moderate |
| An et al. (2013) | 16 | 1.120 | 0.513 | Golf swing | Low |
| Beck et al. (2017) | 32 | 0.161 | 0.173 | Ground walking | Low |
| Becker & Smith (2013) | 24 | -0.271 | 0.275 | Pedalo riding | Moderate |
| Becker & Smith (2013) | 24 | 0.352 | 0.274 | Pedalo riding | Moderate |
| Brocken et al. (2016) | 60 | 0.259 | 0.253 | Golf putting | Low |
| Chiviacowsky et al. (2010) | 32 | 1.039 | 0.368 | Balance | Low |
| Chiviacowsky et al. (2013) | 24 | 1.356 | 0.440 | Beanbag tossing | Moderate |
| Chow et al. (2014) | 16 | -0.245 | 0.475 | Treadmill running | Low |
| Christina & Alpenfels (2014 Exp 1 & 2) | 56 | 0.981 | 0.279 | Golf swing | Low |
| da Silva et al. (2017) | 38 | 1.493 | 0.361 | Ballet pirouette | Moderate |
| Emanuel et al. (2008) | 32 | -0.296 | 0.347 | Dart throwing | Moderate |
| Emanuel et al. (2008) | 34 | 0.369 | 0.338 | Dart throwing | Moderate |
| Gredin & Williams (2016) | 24 | 0.530 | 0.403 | Soccer chipping | Moderate |
| Hadler et al. (2014) | 30 | 1.034 | 0.380 | Tennis serve | Moderate |
| Jackson & Holmes (2011) | 18 | 0.131 | 0.321 | Balance | Low |
| Kim, Hinojosa et al. (2017) | 30 | 0.016 | 0.355 | Arm movement | Low |
| Koufou et al. (2013) | 20 | 0.791 | 0.446 | Forward tuck roll | Moderate |
| Land et al. (2014) | 20 | 1.695 | 0.505 | Golf putting | Low |
| Laufer et al. (2007) | 40 | 0.198 | 0.220 | Balance | Low |
| Lawrence et al. (2011) | 20 | 0.948 | 0.454 | Gymnastics | Low |
| McNevin et al. (2003) | 20 | 0.430 | 0.434 | Balance | Low |
| Mullen et al. (2012) | 16 | 1.161 | 0.515 | Simulation driving | Low |
| Parr & Button (2009) | 12 | 1.585 | 0.623 | Rowing | Low |
| Perreault & French (2015) | 28 | 2.207 | 0.471 | Modified basketball free | Low |
| Saemi et al. (2013) | 20 | 1.281 | 0.474 | Tennis ball throwing | Moderate |
| Samsudin & Low (2017) | 10 | 4.428 | 1.143 | Boule throwing | Low |
| Shafizadeh, Platt & Bahram (2013) | 24 | 0.536 | 0.402 | Dart throwing | Moderate |
| Shea & Wulf (1999) | 32 | 0.768 | 0.350 | Balance | Low |
| Tse & van Ginneken (2017) | 40 | 0.155 | 0.341 | Dart throwing | Low |
| Tse (2017) | 44 | -1.782 | 0.352 | Beanbag throwing | Moderate |
| Tsetseli et al. (2016) | 40 | 0.507 | 0.315 | Tennis match | Moderate |
| Tsetseli et al. (2018) | 44 | 2.259 | 0.382 | Tennis serve | Moderate |
| Uehara et al. (2008) | 12 | -0.294 | 0.536 | Soccer chipping | Moderate |
| Welling et al. (2016) | 20 | 0.090 | 0.429 | Jumping | Low |
| Wulf & Su (2007 Exp 1) | 20 | 0.358 | 0.432 | Golf pitch shot | Low |
| Wulf et al. (1998 Exp 1 & 2) | 38 | 1.371 | 0.351 | Balance | Low |
| Wulf et al. (1999) | 22 | 1.490 | 0.468 | Golf pitch shot | Moderate |
| Wulf et al. (2001) | 28 | 2.007 | 0.455 | Balance | Low |
| Wulf et al. (2002 Exp 1 & 2) | 100 | 1.281 | 0.217 | Volleyball serve, soccer lofted | Moderate |
| Wulf, Chiviacowsky, et al. (2010) | 48 | 0.421 | 0.286 | Soccer throw-in | Low |
| Wulf, Weigelt, et al. (2003) | 38 | 1.688 | 0.367 | Balance | Low |
| Wulf, Wächter, et al. (2003) | 40 | 0.338 | 0.316 | Soccer straight | Low |
| Zentgraf & Munzert (2009) | 46 | 0.181 | 0.290 | Juggling | Low |
| Ziv & Lidor (2015) | 48 | 0.068 | 0.284 | Golf putting | Low |

## Table S3: Transfer effect sizes (k = 23, N = 797)

| **Study** | **n** | **g** | **s** | **Task** | **Risk of bias** |
| --- | --- | --- | --- | --- | --- |
| Abdollahipour et al. (2008) | 76 | 0.514 | 0.227 | Soccer dribbling | Moderate |
| Abdollahipour et al. (2014) | 40 | -0.134 | 0.096 | Dart throwing | Moderate |
| Abdollahipour et al. (2019 Exp 2) | 26 | 1.463 | 0.305 | Pedalo riding | Moderate |
| Agar et al. (2016) | 48 | -0.297 | 0.281 | Puck shooting | Low |
| Chiviacowsky et al. (2013) | 24 | 1.090 | 0.424 | Beanbag tossing | Moderate |
| da Silva et al. (2017) | 38 | 1.246 | 0.348 | Ballet pirouette | Moderate |
| Emanuel et al. (2008) | 32 | -0.435 | 0.349 | Dart throwing | Moderate |
| Emanuel et al. (2008) | 34 | 0.514 | 0.341 | Dart throwing | Moderate |
| Flôres et al. (2005) | 54 | 0.423 | 0.189 | Pedalo riding | Moderate |
| Gredin & Williams (2016) | 24 | -0.081 | 0.157 | Soccer chipping | Moderate |
| Hadler et al. (2014) | 30 | 1.279 | 0.392 | Tennis serve | Moderate |
| Kalkhoran & Shariati (2014) | 30 | 1.454 | 0.292 | Basketball dribbling | Moderate |
| Landers et al. (2016) | 21 | -0.013 | 0.297 | Balance | Low |
| Lawrence et al. (2011) | 20 | 0.937 | 0.453 | routine | Low |
| Makaruk et al. (2012) | 24 | 0.651 | 0.237 | Jumping | Low |
| Parr & Button (2009) | 12 | 1.814 | 0.649 | Rowing | Low |
| Shafizadeh, Platt & Bahram (2013) | 24 | 0.746 | 0.409 | Dart throwing | Moderate |
| Totsika & Wulf (2003) | 22 | 0.862 | 0.249 | Pedalo riding | Moderate |
| Tse (2017) | 44 | -1.136 | 0.320 | Beanbag throwing | Moderate |
| Wulf, Chiviacowsky, et al. (2010) | 48 | 0.313 | 0.285 | Soccer throw-in | Low |
| Wulf, Weigelt, et al. (2003) | 38 | 1.410 | 0.351 | Balance | Low |
| Wulf, Wächter, et al. (2003) | 40 | 0.323 | 0.308 | pass | Low |
| Ziv & Lidor (2015) | 48 | -0.123 | 0.284 | Golf putting | Low |

## Table S4: EMG efficiency effect sizes (k = 16, N = 299)

| **Study** | **n** | **g** | **s** | **Task** | **Risk of bias** |
| --- | --- | --- | --- | --- | --- |
| Ducharme & Wu (2015) | 29 | 0.140 | 0.184 | Balance | Low |
| Greig & Marchant (2014) | 25 | 1.502 | 0.171 | Elbow flexion | Low |
| Halperin, Hughes, et al. (2016) | 28 | -0.075 | 0.187 | Elbow flexion | Low |
| Kal et al. (2013) | 30 | -0.112 | 0.181 | Cyclic single-leg extension-flexion | Low |
| Kuhn et al. (2017) | 10 | 1.628 | 0.474 | Finger abduction | Low |
| Kuhn et al. (2018 Exp 1) | 14 | 0.275 | 0.265 | Finger flexion | Low |
| Lohse & Sherwood (2012 Exp 1 & 2) | 24 | 0.419 | 0.147 | Force production (plantar flexion) | Low |
| Lohse et al. (2010) | 12 | 1.370 | 0.395 | Dart throwing | Low |
| Lohse et al. (2011) | 12 | 1.516 | 0.416 | Force production (plantar flexion) | Low |
| Marchant & Greig (2017) | 20 | 0.279 | 0.224 | Force production (knee extension) | Low |
| Marchant, Greig, et al. (2009) | 25 | 1.705 | 0.311 | Elbow flexion | Low |
| Pelleck & Passmore (2017) | 11 | 0.037 | 0.205 | Golf putting | Low |
| Pelleck & Passmore (2017) | 13 | 0.075 | 0.190 | Golf putting | Low |
| Vance et al. (2004 Exp 1 & 2) | 24 | 1.420 | 0.286 | Biceps curls | Low |
| Wulf, Dufek, et al. (2010) | 8 | 1.715 | 0.544 | Jumping | Low |
| Zachry et al. (2005) | 14 | 1.310 | 0.359 | throw | Moderate |

## Table S5: Distance effect effect sizes (k = 12, N = 287)

| **Study** | **n** | **g** | **s** | **Task** | **Risk of bias** |
| --- | --- | --- | --- | --- | --- |
| Coker (2016) | 21 | -0.232 | 0.217 | Jumping | Moderate |
| Duke et al. (2011) | 12 | 0.812 | 0.324 | Piano playing | Low |
| Duke et al. (2011) | 4 | 0.374 | 0.454 | Piano playing | Low |
| Kearney (2015) | 18 | 0.873 | 0.273 | Golf putting | Low |
| Lohse et al. (2014) | 15 | 5.248 | 0.991 | Dart throwing | Low |
| Marchant, Griffiths, et al. (2018) | 54 | 0.101 | 0.135 | Jumping | Moderate |
| McKay & Wulf (2012) | 36 | 0.417 | 0.172 | Dart throwing | Moderate |
| Porter et al. (2012) | 35 | 0.191 | 0.169 | Jumping | Moderate |
| Porter et al. (2013) | 38 | 0.335 | 0.165 | Jumping | Moderate |
| Raisbeck & Yamada (2019) | 24 | 0.291 | 0.205 | Jumping | Low |
| Stambaugh (2017) | 15 | -0.461 | 0.265 | Playing of musical instrument | Low |
| Stambaugh (2017) | 15 | 0.017 | 0.251 | Playing of musical instrument | Low |

# References

Chua, L. K., Dimapilis, M. K., Iwatsuki, T., Abdollahipour, R., Lewthwaite, R., & Wulf, G. (2021). Practice for agility: A systematic review and meta-analysis of attentional focus effects. Psychological Bulletin, 147(11), 1145–1168.

Hedges, L. V. (1981). Distribution theory for Glass's estimator of effect size and related estimators. Journal of Educational Statistics, 6(2), 107–128.

Higgins, J. P., & Thompson, S. G. (2002). Quantifying heterogeneity in a meta-analysis. Statistics in Medicine, 21(11), 1539–1558.

McKay, B., Lewthwaite, R., & Wulf, G. (2024). Attentional focus effects in motor learning and performance: An updated meta-analysis. Public repository.
